# A comparison of blood and brain‐derived ageing and inflammation‐related DNA methylation signatures and their association with microglial burdens

**DOI:** 10.1111/ejn.15661

**Published:** 2022-04-06

**Authors:** Anna J. Stevenson, Daniel L. McCartney, Danni A. Gadd, Gemma Shireby, Robert F. Hillary, Declan King, Makis Tzioras, Nicola Wrobel, Sarah McCafferty, Lee Murphy, Barry W. McColl, Paul Redmond, Adele M. Taylor, Sarah E. Harris, Tom C. Russ, Andrew M. McIntosh, Jonathan Mill, Colin Smith, Ian J. Deary, Simon R. Cox, Riccardo E. Marioni, Tara L. Spires‐Jones

**Affiliations:** ^1^ Centre for Genomic and Experimental Medicine, Institute of Genetics and Molecular Medicine University of Edinburgh Edinburgh UK; ^2^ Centre for Discovery Brain Sciences University of Edinburgh Edinburgh UK; ^3^ University of Exeter Medical School University of Exeter Exeter UK; ^4^ UK Dementia Research Institute University of Edinburgh Edinburgh UK; ^5^ Edinburgh Clinical Research Facility Western General Hospital Edinburgh UK; ^6^ Lothian Birth Cohorts University of Edinburgh Edinburgh UK; ^7^ Department of Psychology University of Edinburgh Edinburgh UK; ^8^ Alzheimer Scotland Dementia Research Centre, 7 George Square University of Edinburgh Edinburgh UK; ^9^ Division of Psychiatry University of Edinburgh, Royal Edinburgh Hospital Edinburgh UK; ^10^ Centre for Clinical Brain Sciences University of Edinburgh Edinburgh UK

**Keywords:** CRP, DNA methylation, epigenetic clock, IL‐6, inflammation, microglia

## Abstract

Inflammation and ageing‐related DNA methylation patterns in the blood have been linked to a variety of morbidities, including cognitive decline and neurodegenerative disease. However, it is unclear how these blood‐based patterns relate to patterns within the brain and how each associates with central cellular profiles. In this study, we profiled DNA methylation in both the blood and in five post mortem brain regions (BA17, BA20/21, BA24, BA46 and hippocampus) in 14 individuals from the Lothian Birth Cohort 1936. Microglial burdens were additionally quantified in the same brain regions. DNA methylation signatures of five epigenetic ageing biomarkers (‘epigenetic clocks’), and two inflammatory biomarkers (methylation proxies for C‐reactive protein and interleukin‐6) were compared across tissues and regions. Divergent associations between the inflammation and ageing signatures in the blood and brain were identified, depending on region assessed. Four out of the five assessed epigenetic age acceleration measures were found to be highest in the hippocampus (β range = 0.83–1.14, *p* ≤ 0.02). The inflammation‐related DNA methylation signatures showed no clear variation across brain regions. Reactive microglial burdens were found to be highest in the hippocampus (β = 1.32, *p* = 5 × 10^−4^); however, the only association identified between the blood‐ and brain‐based methylation signatures and microglia was a significant positive association with acceleration of one epigenetic clock (termed DNAm PhenoAge) averaged over all five brain regions (β = 0.40, *p* = 0.002). This work highlights a potential vulnerability of the hippocampus to epigenetic ageing and provides preliminary evidence of a relationship between DNA methylation signatures in the brain and differences in microglial burdens.

## Graphical abstract

DNA methylation was profiled in the blood and in five post mortem brain regions. Microglial burdens were quantified in the same brain regions. DNA methylation signatures of five epigenetic ageing biomarkers (‘epigenetic clocks’), and two inflammatory biomarkers (methylation proxies for CRP and IL‐6) were compared across tissues and regions to investigate the link between peripheral and central inflammation‐ and age‐related methylation patterns and how they relate to central cellular profiles.

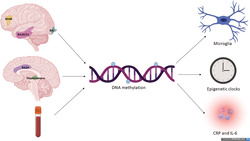

**List of Abbreviations**
BA: Brodmann area
BBB: blood brain barrier
CD68: cluster of differentiation 68
CRP: C‐reactive protein
DAB: 3,3′‐diaminobenzidine
DNA: deoxyribonucleic acid
DNAm: DNA methylation
EEAA: extrinsic epigenetic age acceleration
EWAS: epigenome wide association study
IEAA: intrinsic epigenetic age acceleration
IL‐6: interleukin‐6
LBC1936: Lothian Birth Cohort 1936
SD: standard deviation
SE: standard error
SNP: single nucleotide polymorphism
TBS: tris‐buffered saline

## 1 INTRODUCTION

Ageing is characterised by a progressive deterioration of physiological integrity and is a key risk factor for a multitude of diseases. A pervasive feature of ageing is a persistent, or chronic, systemic inflammation (Franceschi et al., 2000). This process is characterised by a subtle elevation of inflammatory mediators in the periphery, in the absence of evident precipitants or disease states. Chronic inflammation has been identified as a common feature in the preponderance of neurodegenerative diseases and is increasingly recognised as a potential mediator of cognitive impairment in older age (Amor et al., 2010). There is, however, still a lack of understanding of the biological mechanisms involved in chronic inflammation and how peripheral and central inflammatory mechanisms relate.

Recently, the link between inflammation and the epigenetic mechanism of DNA methylation (DNAm) has begun to be addressed (Gonzalez‐Jaramillo et al., 2019; Ligthart et al., 2016). DNAm is typically characterised by the addition of a methyl group to a cytosine, in the context of a cytosine‐guanine (CpG) dinucleotide. It has been implicated in the regulation of gene expression and can itself be influenced by both genetic and environmental factors (Beck & Rakyan, 2008; Jaenisch & Bird, 2003). Genome‐wide DNAm patterns in the blood have been leveraged to index lifestyle traits, such as smoking (Liu et al., 2018; McCartney et al., 2018), and have been used to investigate diverse physical and mental health‐related phenotypes, including cognitive functioning (McCartney et al., 2022). In addition to this, by exploiting the manifest alterations in DNAm patterns with ageing, several DNAm‐based markers of age have been developed, which attempt to provide surrogate measures of biological ageing (Hannum et al., 2013; Horvath, 2013; Levine et al., 2018; Lu et al., 2019). These ‘epigenetic clocks’ have been used to provide a measure of biological age acceleration, or deceleration, by establishing the difference between an individual's chronological and epigenetic age. Positive age acceleration quantified in the blood has been associated with an increased risk of mortality and a variety of age‐related morbidities, including with a lower cognitive ability (Beydoun et al., 2020; Hillary et al., 2019; Marioni et al., 2015). In addition to this, we found that blood‐based DNAm proxies for two inflammatory mediators—C‐reactive protein (CRP) and interleukin‐6 (IL‐6)—were inversely associated with cognitive ability in older adults with larger effect sizes compared with the biomarkers themselves (Stevenson et al., 2020, 2021).

While these findings suggest that an accelerated biological age, and raised DNAm inflammation patterns associate with poorer cognitive functioning, it is important to note that these studies analysed blood tissue. While the blood represents a practical, accessible source by which to investigate such outcomes, DNAm is known to confer both cell‐type and tissue‐specific patterns (Mendizabal & Yi, 2016). For analyses of brain‐based traits such as cognitive ability, brain samples offer the optimal disease‐relevant tissue; however, given the obvious limitations of access to such tissue, much of the research assessing the association between differential DNAm and disorders of the central nervous system has been conducted in peripheral whole blood (Chuang et al., 2017; Di Francesco et al., 2015). While this approach can provide informative peripheral markers of central aberration or disease, it is important to investigate the relevant target tissue to characterise both how peripheral and central patterns equate, and how each relates to cellular differences within the brain. Microglia are the primary tissue‐resident immune cells of the central nervous system and have critical roles in homeostasis and neuroinflammation. Aged microglia have been shown to be more responsive to pro‐inflammatory stimuli compared with naïve microglia, and evidence suggests the cells are particularly sensitive to both acute and chronic systemic inflammation detected via peripheral‐central signalling pathways (Cunningham et al., 2005; Norden & Godbout, 2013). Microglia have additionally been implicated in age‐related neurological dysfunction (Kaneshwaran et al., 2019; Luo et al., 2010; Norden & Godbout, 2013). However, as yet, it is unclear how inflammation and age‐related DNAm patterns in both the periphery and the brain itself relate to microglial burdens.

In this study, we utilise data from 14 participants of the Lothian Birth Cohort 1936. These individuals have blood‐based DNAm data available at up to 4 time‐points between the ages of 70–79 years and additionally donated post mortem brain tissue to the study. In the brain, we profiled DNAm and quantified microglial burdens in five regions (inferior temporal gyrus [BA20/21], ventral anterior cingulate cortex [BA24], dorsolateral prefrontal cortex [BA46], hippocampus and primary visual cortex [BA17]). The first four regions were selected as they are typically implicated in neurodegenerative diseases such as Alzheimer's disease. Conversely, BA17 is relatively spared from pathology until the latter stages of disease pathogenesis and was thus chosen to act as an internal control (Cui et al., 2007; Keller, 2006). DNAm CRP and IL‐6 profiles, along with five different DNAm age acceleration measures, were characterised in the blood and in each brain region to investigate the relationship between peripheral and central age‐ and inflammation‐related methylation patterns and how these relate to inflammatory processes in the brain. Given the small sample size of this study, the results presented here represent preliminary patterns. However, these data, and the methodology employed, provide a framework upon which future larger scale work can be based.

## 2 METHODS

### 2.1 The Lothian Birth Cohort 1936

The Lothian Birth Cohort 1936 (LBC1936) is a longitudinal study of ageing. Full details on the study protocol and data collection have been described previously (Deary et al., 2007; Taylor et al., 2018). Briefly, the cohort comprises 1091 individuals born in 1936 most of whom completed a study of general intelligence—the Scottish Mental Survey—in 1947 when they were aged around 11 years. Participants who were living in Edinburgh and the surrounding area were re‐contacted around 60 years later with 1091 individuals consenting to join the LBC1936 study. At Wave 1 of the study, participants were around 70 years old (mean age: 69.6 ± 0.8 years), and they have since completed up to four additional assessments, triennially. At each assessment, participants have been widely phenotyped with detailed physical, cognitive, epigenetic, health and lifestyle data collected. A tissue bank for post mortem brain tissue donation was established at Wave 3 of LBC1936 in collaboration with the Medical Research Council‐funded University of Edinburgh Brain Banks. To date, ∼15% of the original LBC1936 sample have given consent for post mortem tissue collection. At the time of this study, samples from 14 individuals were available.

### 2.2 Ethics

Ethical permission for LBC1936 was obtained from the Multi‐Centre Research Ethics Committee for Scotland (MREC/01/0/56), the Lothian Research Ethics Committee (Wave 1: LREC/2003/2/29) and the Scotland A Research Ethics Committee (Waves 2, 3 and 4: 07/MRE00/58).

Use of human tissue for post mortem studies was reviewed and approved by the Edinburgh Brain Bank ethics committee and the medical research ethics committee (the Academic and Clinical Central Office for Research and Development, a joint office of the University of Edinburgh and NHS Lothian, approval number 15‐HV‐016). The Edinburgh Brain Bank is a Medical Research Council funded facility with research ethics committee (REC) approval (16/ES/0084).

### 2.3 DNA methylation preparation

#### 2.3.1 Blood

DNAm from whole blood was quantified at 485,512 CpG sites using the Illumina Human Methylation 450k BeadChips at the Edinburgh Clinical Research Facility. Full details of the quality control steps have been described previously (Shah et al., 2014; Zhang et al., 2018). Briefly, raw intensity data were background‐corrected and normalised using internal controls. Samples with inadequate bisulphite conversion, hybridisation, staining signal or nucleotide extension were removed upon manual inspection. Further, probes with a low detection rate (*p* > 0.01 in >5% of samples), samples with a low call rate (<450,000 probes detected at *p* < 0.01), samples exhibiting a poor match between genotype and SNP control probes and samples with a mismatch between methylation‐predicted and recorded sex were additionally excluded. This left a total of 450,276 autosomal probes. In analyses comparing blood and brain DNAm signatures, the last blood measurement before death was used and models were adjusted for the interval between the blood draw and death (see Table S1; mean interval: 2.5 years, SD: 1.5).

#### 2.3.2 Brain

Brains were removed at post mortem and cut into coronal slices. Regions of interest were dissected, as detailed previously (Samarasekera et al., 2013). Tissue samples from cortical regions BA17, BA20–21, BA24, BA46 and hippocampus were collected and snap frozen. From these sections, ~25 mg of tissue was processed for DNA extraction. DNA extraction was performed using a DNeasy kit (Qiagen) and DNAm was profiled using Illumina MethylationEPIC BeadChips at the Edinburgh Clinical Research Facility. Samples were processed randomly. Quality control steps were performed as follows: The *wateRmelon* pfilter() function (Pidsley et al., 2013) was used to remove samples in which >1% of probes had a detection *p* value of >0.05, probes with a beadcount of <3 in >5% of samples and probes in which >1% of samples had a detection *p* value of >0.05. Probes mapping to polymorphic targets, cross‐hybridising probes and probes on the X and Y chromosomes were additionally removed. The performance of 15 normalisation functions was assessed, following the protocol described by Pidsley et al. (Pidsley et al., 2013). The top‐ranking method was *danet* which equalises background from type 1 and type 2 probes, performs quantile normalisation of methylated and un‐methylated intensities simultaneously, and then calculates normalised methylation β‐values. The normalised dataset comprised 69 samples (14 individuals, 5 regions, 1 missing hippocampal sample) and 807,163 probes.

### 2.4 Derivation of DNA methylation signatures

#### 2.4.1 Epigenetic age acceleration

Methylation‐based epigenetic age acceleration estimates were obtained from the online Horvath DNAm age calculator (https://dnamage.genetics.ucla.edu/) (Horvath, 2013). Normalised DNAm data were uploaded to the calculator using the ‘Advanced Analysis’ option. This output provides four different age acceleration measures: intrinsic epigenetic age acceleration (IEAA) (Horvath, 2013); extrinsic epigenetic age acceleration (EEAA) (Hannum et al., 2013); DNAm PhenoAge acceleration (AgeAccel_Pheno_) (Levine et al., 2018); and DNAm GrimAge acceleration (AgeAccel_Grim_) (Lu et al., 2019). IEAA is defined as the residuals resulting from the regression of estimated epigenetic age based on the Horvath epigenetic clock on chronological age, fitting estimated proportions of immune cells. IEAA is designed to capture cell‐intrinsic epigenetic ageing, independent of age‐related changes in blood cellular composition. EEAA is estimated firstly by calculating a weighted average of Hannum's methylation age with three cell types—naïve cytotoxic T cells, exhausted cytotoxic T cells and plasmablasts. EEAA is defined as the residuals resulting from the univariate regression of this weighted estimate on chronological age and correlates with age‐related changes in the blood cellular composition. Though these measures are most appropriate for use in the blood as they account for blood cell proportions, the correlation between these and the unadjusted measures are high (0.78 for IEAA‐HorvathAgeAccel and 0.97 for EEAA‐HannumAgeAccel), suggesting they are very similar. Rather than aiming to predict chronological age, DNAm PhenoAge was designed to capture an individual's ‘phenotypic age’—a composite set of clinical measures associated with mortality. Regressing DNAm PhenoAge onto chronological age provides the acceleration measure: AgeAccel_Pheno_. Similarly, DNAm GrimAge was designed to predict mortality based on a linear combination of age, sex, and DNAm‐based surrogates for smoking and seven plasma proteins. AgeAccel_Grim_ provides the measure of epigenetic age acceleration from this clock. In addition to the epigenetic age acceleration measures, the online calculator provides an estimate of the proportion of neurons in each sample, derived using the cell epigenotype specific (CETS) algorithm (Guintivano et al., 2013).

Recently, an epigenetic clock (DNAmClock_Cortical_) was developed to optimally capture brain‐specific epigenetic ageing (Shireby et al., 2020). This clock was trained on nine human cortex methylation datasets of tissue from individuals unaffected by Alzheimer's disease (total *n* = 1397, age range = 1–104 years). The model selected 347 DNAm sites, and the clock was then tested in an external cohort, outperforming other epigenetic clocks for age prediction within the brain. The sum of DNAm levels at these sites weighted by their regression coefficients provided the cortical DNAmClock_Cortical_ age estimate. The residuals resulting from regressing DNAmClock_Cortical_ age on chronological age provided the age acceleration measure for this epigenetic clock (AgeAccel_Cortical_).

#### 2.4.2 Inflammation signatures

DNAm scores for the acute‐phase inflammatory mediator C‐reactive protein (CRP) and the pro‐inflammatory cytokine interleukin‐6 (IL‐6) were derived as described previously (Barker et al., 2018; Stevenson et al., 2020, 2021). The DNAm CRP score was obtained using data from a large epigenome‐wide association study (EWAS) of CRP (Ligthart et al., 2016). This EWAS identified seven CpG sites with strong evidence of a functional association with circulating CRP. One of these CpGs (cg06126421) was not available on the EPIC array; therefore, the sum of DNAm levels at the remaining six CpG sites weighted by their regression coefficients from the EWAS provided the DNAm CRP score (Barker et al., 2018) (Table S2). The IL‐6 score was derived from an elastic net penalised regression model using the Wave 1 LBC1936 blood methylation and Olink® IL‐6 data (Olink® inflammation panel, Olink® Bioscience, Uppsala, Sweden) (Stevenson et al., 2021). This approach identified 35 CpG sites that optimally predicted circulating IL‐6. In the current study, the elastic net regression was re‐run omitting individuals providing post mortem brain samples (*n* = 863). This model returned a set of 34 CpG sites (28 CpGs common to both models, 0.91 correlation with original score). The DNAm IL‐6 score in both blood and brain were thus derived from the sum of DNAm levels at these 34 CpG sites weighted by their regression coefficients (Table S3).

#### 2.4.3 Immunohistochemistry, thresholding and burden quantification

Tissue samples were resected from post mortem brains, dehydrated with ethanol and processed for paraffin embedding. Fixed tissue sections from cortical regions BA17, BA20–21, BA24, BA46 and hippocampus were cut using a microtome (4 μm) and processed for immunohistochemistry. Paraffin‐embedded sections were dewaxed in xylene and rehydrated through graded ethanol solutions. Immunohistochemistry was performed using standard protocols, enhanced with the Novolink Polymer Detection Kit. Briefly, antigen retrieval was performed in citric acid in a pressure cooker. Sections were then washed in dH_2_0 (5 min), followed by a peroxidase block (30 min), a Tris‐buffered saline (TBS) wash (5 min), a protein block (15 min) and another TBS wash (5 min). CD68 antibody (mouse anti‐human monoclonal primary antibody, Dako M0876, 1:100) was applied (30 min at room temperature), followed by post primary block (30 min), TBS wash (5 min), Novolink Polymer incubation (30 min) and a final TBS wash (5 min). 3,3′‐Diaminobenzidine (DAB) with 0.05% hydrogen peroxide as chromogen was used for visualisation. Tissue was counterstained with haematoxylin for 30 s to visualise cell nuclei. Finally, sections were dehydrated through a series of ethanol solutions and xylene, and coverslips were mounted.

Stains were visualised using a ZEISS Imager.Z2 stereology microscope using MBF Biosciences Stereo Investigator software. All six layers of cortical grey matter were included in analysis. Cortical grey matter was outlined at 1.5× objective magnification, and tile scans were acquired at 5× for quantification. Glia were quantified using in‐built software that captures immuno‐positive objects using an automated thresholding algorithm based on colour and size. Objects smaller than 10 μm^2^ were not considered true staining and were thus excluded in the burden analysis. The threshold and exposure remained consistent throughout all analysis. Neurolucida Explorer was used to quantify the total area of the region of interest and that of the outlined objects. A percentage burden was then calculated by dividing the stained area by the total tissue area.

### 2.5 Statistical analyses

Spearman correlations were calculated between the inflammation and epigenetic age acceleration measures in the blood and the brain, and between the blood and each brain region using the last available blood‐based measure prior to death. Linear mixed effects models were used to investigate the regional heterogeneity in the epigenetic age acceleration variables and the DNAm inflammation scores in the brain. BA17 was set as the reference as this region is typically not affected until the latter stages of neurodegenerative diseases that impact cognitive functioning, such as Alzheimer's disease. Models were adjusted for age at death, post mortem interval, sex and proportion of neurons, with participant ID fitted as a random effect on the intercept. Linear mixed effects models were additionally used to assess the association between the DNAm signatures in both the blood and the brain and CD68^+^ microglial burdens. Here, an interaction term between the brain region and DNAm score was included to test if any effects were region dependent. The same covariates and random effect as above were included. Models assessing blood‐based signatures were additionally adjusted for the interval between their measurement and death. In each regression analysis, continuous variables were scaled to have a mean of zero and unit variance. We considered a statistical significance threshold of *p* < 0.05. We additionally discuss how results change at a more conservative Bonferroni‐corrected level of significance (*p* < 0.05/41 = 0.001).

## 3 RESULTS

### 3.1 Cohort demographics

Post mortem details for each individual included in the study are presented in Table S1. Summary statistics for each of the variables included in analyses is presented in Table 1. Age at death ranged from 77.6 to 82.9 years (mean = 80.3, SD = 1.56). Five of the 14 (36%) individuals were female.

**TABLE 1:** Summary of the variables assessed in the 14 Lothian birth cohort 1936 participants

Variable	Mean	SD
Sex (% female)	35.71	‐
Age at death (years)	80.33	1.56
Age at last blood draw	77.88	1.67
Brain		
DNAm CRP score	−0.014	6.1 × 10^−4^
DNAm IL‐6 score	0.016	0.0045
AgeAccel_Cortical_	−0.52	6.12
AgeAccel_Grim_	−0.31	2.32
AgeAccel_Pheno_	0.053	5.71
IEAA	−0.049	3.97
EEAA	−0.55	3.38
CD68 burden (%)	0.34	0.38
Blood		
DNAm CRP score	−0.014	1.2 × 10^−3^
DNAm IL‐6 score	0.020	6.5 × 10^−3^
AgeAccel_Grim_	6.68	6.53
AgeAccel_Pheno_	3.23	8.48
IEAA	1.23	5.62
EEAA	2.99	11.20

*Note*: The brain variables refer to the mean across all five regions.

Abbreviations: CD68, Cluster of Differentiation 68 (microglial burden); CRP, C‐reactive protein; DNAm, DNA methylation; EEAA, extrinsic epigenetic age acceleration; IEAA, intrinsic epigenetic age acceleration; IL‐6, interleukin‐6.

### 3.2 DNAm inflammation signatures

The Spearman correlation between the last blood DNAm CRP score and the mean brain DNAm CRP score was 0.061. This blood–brain correlation varied by region, ranging from −0.52 in BA17 to 0.46 in BA46 (Figure S1).

**FIGURE 1:**
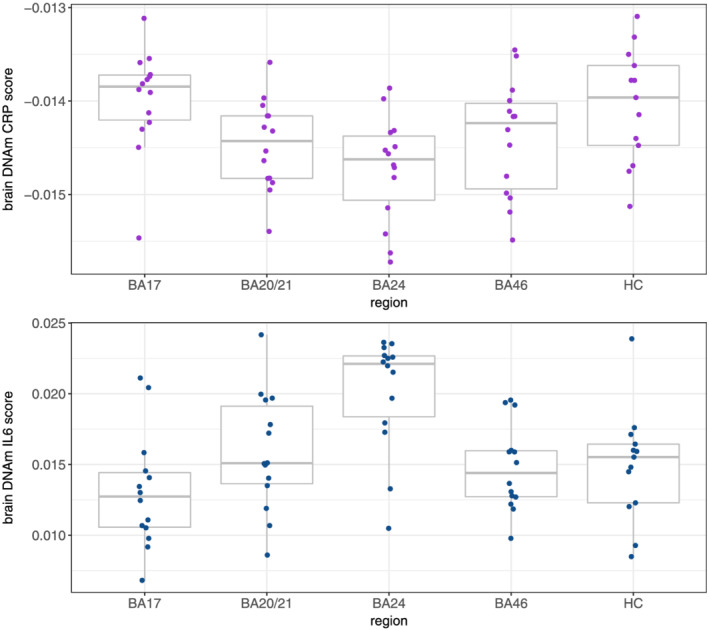
The DNAm CRP and IL‐6 score in each of the five regions of the brain. Abbreviations: BA, Brodmann area; CRP, C‐reactive protein; DNAm, DNA methylation; HC, hippocampus; IL‐6, interleukin‐6

A boxplot of the DNAm CRP score in the five brain regions is presented in Figure 1. No significant differences were identified in the analysis by region (Table S4), indicating none of the assessed regions had a significantly different DNAm CRP score compared with BA17 (reference region).

The correlation between the last blood DNAm IL‐6 score and the mean brain DNAm IL‐6 score was 0.045, ranging from −0.33 in the hippocampus to 0.25 in BA24 (Figure S2).

A boxplot of the DNAm IL‐6 score in the five brain regions is presented in Figure 1. In the analysis by region, the DNAm IL‐6 score was found to be significantly higher in BA24 (β = 0.46, *SE* = 0.20, *p* = 0.024) compared with BA17 (reference region, Table S4).

### 3.3 DNAm age acceleration

The correlations between the last blood DNAm age acceleration and the mean age acceleration in the brain were −0.04 for IEAA, 0.48 for EEAA, 0.39 for AgeAccel_Grim_ and 0.30 AgeAccel_Pheno_. Correlation plots between the last blood DNAm age acceleration measure and the DNAm age acceleration in the brain split by region are presented in Figures S3–S6. The coefficients for AgeAccel_Grim_, AgeAccel_Pheno_ and EEAA were all positive, ranging from 0.09 between AgeAccel_Pheno_ in the blood and in BA46 to 0.78 between the last blood EEAA and EEAA in BA17. IEAA showed a negative correlation between the last blood measurement and the measure in BA20/21 (*r* = −0.27), BA24 (*r* = −0.14) and BA46 (*r* = −0.25) but a positive correlation in the hippocampus (*r* = 0.30) and BA17 (*r* = 0.49). For EEAA, some of the positive correlations appear largely driven by an individual with a high last blood measure (38.2) which corresponded with high measures in each of the brain regions (Figure S4). This individual additionally had consistently high last blood measures in each of the other epigenetic age acceleration measures assessed (range: 6.6–25.4).

**FIGURE 2:**
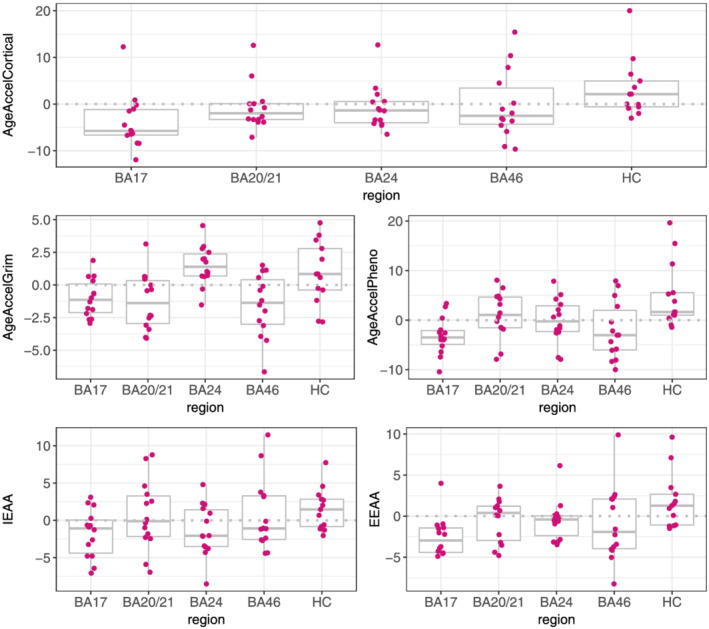
DNAm age acceleration measures across the five brain regions. The dashed grey lines represent where the mean difference is zero. Abbreviations: BA, Brodmann area; EEAA, extrinsic epigenetic age acceleration; HC, hippocampus; IEAA, intrinsic epigenetic age acceleration

Boxplots of the five different epigenetic age acceleration measures in each of the five brain regions are presented in Figure 2. The hippocampus displayed the highest DNAm age acceleration compared with BA17 (reference region) for each of the assessed measures except for AgeAccel_Grim_ which was highest in BA24 (Table S5; AgeAccel_Cortical_: β = 0.901, *SE* = 0.19, *p* = 2.6 × 10^−5^; AgeAccel_Pheno_: β = 1.14, *SE* = 0.27, *p* = 1 × 10^−4^; IEAA: β = 0.83, *SE* = 0.34, *p* = 0.02; EEAA: β = 0.99, *SE* = 0.24, *p* = 1 × 10^−4^). The result for EEAA remained similar when the individual with consistently high measures across all regions was removed (β = 1.22, *SE* = 0.30, *p* = 1.4 × 10^−4^).

### 3.4 Inter‐tissue correlations

The Spearman correlations between the last blood DNAm age acceleration and inflammation measures are presented in Figure S7. Here, all but two of the coefficients (DNAm CRP score‐AgeAccel_Grim_ [*r* = −0.09] and DNAm CRP score‐IEAA [*r* = −0.14]) were positive, suggesting the both the age acceleration measures and inflammation scores are largely correlated within the blood.

The Spearman correlations between the mean brain DNAm age acceleration and inflammation measures are presented in Figure S8. Discounting the DNAm CRP score, and similarly to the blood, all but two of the coefficients (DNAm IL‐6 score‐IEAA [*r* = −0.02] and AgeAccel_Grim_‐IEAA [*r* = −0.18]) were positive. The DNAm CRP score showed a consistent negative correlation with all other measures excepting a small positive correlation with AgeAccel_Grim_ (*r* = 0.014).

### 3.5 Microglial burdens

**FIGURE 3:**
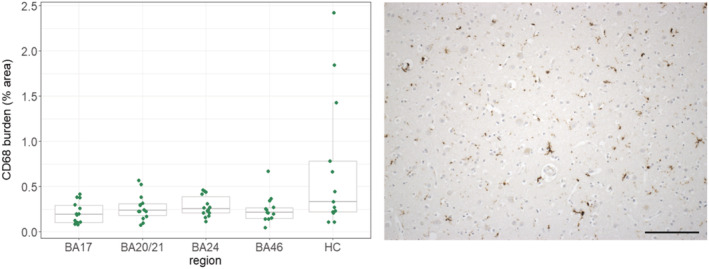
CD68^+^ microglial burdens over the five brain regions and representative staining (BA46). Abbreviations: BA, Brodmann area; HC, hippocampus. Scale bar = 150 μm

A boxplot of the CD68^+^ microglial burdens in each of the five brain regions and a representative imaging of the staining is presented in Figure 3 (exemplar images of the staining in each of the brain regions analysed are presented in Figure S9). The microglial burden was found to be significantly higher in the hippocampus compared with BA17 (β = 1.32, *SE* = 0.4, *p* = 5 × 10^−4^), with the plot suggesting large variance in this region compared with the others.

The associations between both the DNAm age acceleration variables and the DNAm inflammation signatures with microglial burdens are presented in Table S6. Here, a higher mean AgeAccel_Pheno_ in the brain associated with an increased microglial burden (β = 0.40, *SE* = 0.14, *p* = 0.002). No other significant associations were identified (all *p* ≥ 0.1), and there were no significant interactions found between any of the methylation scores and brain region.

## 4 DISCUSSION

In this study, we took advantage of blood and post mortem brain tissue available in 14 individuals in LBC1936 to investigate the relationship between peripheral and central inflammation‐ and age‐related DNAm signatures and how they relate to neuroinflammatory processes. Due to the small sample size the results of this work are preliminary; however, some potentially interesting patterns were identified. We found heterogeneous correlations between both the age acceleration, and inflammation‐related, methylation signatures in the blood and the brain depending on the region assessed. Of the inflammatory signatures, the DNAm CRP score did not show significant variation across the brain regions, while the DNAm IL‐6 score was found to be higher in BA24. Other than for AgeAccel_Grim_, epigenetic age acceleration was found to be significantly higher in the hippocampus than in BA17. Reactive microglial burdens, identified through CD68 immunostaining, were additionally found to be higher in the hippocampus, consistent with previous findings in a smaller sample of the LBC1936 cohort (Tzioras et al., 2017). However, the only association identified between the DNAm signatures (age acceleration or inflammation proxies) and microglial load was a positive association with the mean brain‐based DNAm AgeAccel_Pheno_.

It is recognised that DNAm patterns at individual CpG sites in the blood and the brain are often disparate (Hannon et al., 2015). We found that DNAm scores for CRP and IL‐6 comprising multiple CpG sites displayed heterogeneous, region‐specific correlations when comparing the blood‐ and brain‐derived signatures. This suggests that blood DNAm patterns may proxy methylation in some areas of the brain better than others. Additionally, it cautions against the use of a single sample of post mortem brain tissue as representative of the brain in aggregate, as it appears there is additional heterogeneity in methylation patterns even within the same tissue source. The DNAm age acceleration measures additionally displayed discrepant blood–brain correlations dependant on region. However, all the assessed measures showed positive blood–brain correlations in each region, to a greater or lesser degree, excepting IEAA. IEAA is based on the Horvath clock which is regarded as a pan‐tissue model (Horvath, 2013), whereas the other three peripheral measures were derived solely on blood DNAm data. Estimates from the Horvath clock have previously been found to be consistent across tissue types, making it surprising that IEAA showed the most inconsistent blood–brain correlation. A recent study has, however, suggested that the age prediction ability of the Horvath clock begins to deteriorate in older age (>60 years), possibly due to saturation of methylation levels at some loci (El Khoury et al., 2019). This may have impacted our results given both blood and brain tissue were gathered from 70 years onwards. The blood–brain correlations identified here suggest significant heterogeneity between the tissues, contingent on region; however, it should be noted that the mean interval between methylation assessed in the blood and in the brain was 2.5 years (range: 4 months–6 years) which reflects a period where methylation alterations are possible (Zaimi et al., 2018).

Within the blood, the DNAm inflammation and age acceleration measures were largely positively correlated, with only a couple of small negative correlations identified. This was likewise true within the brain; however, here the DNAm CRP score displayed a consistent negative association with each measure except AgeAccel_Grim_. In the regional analyses of DNAm signatures in the brain, no notable differences emerged in the assessment of the DNAm CRP score. On the other hand, the DNAm IL‐6 score seemed to be higher in BA24 compared with BA17, possibly suggesting a disparity in the DNAm inflammation signatures across the brain. CRP itself does not typically cross the blood–brain barrier (BBB) although its pro‐inflammatory effects may lead to an increased paracellular permeability of the BBB (Elwood et al., 2017). Conversely, IL‐6 can cross the BBB through the brain's circumventricular organs and is additionally expressed in the brain itself. However, the DNAm signatures of CRP and IL‐6 were both created in blood and have not yet been validated in brain tissue. Work to assess other blood‐calibrated predictors within in brain tissue is currently ongoing (Gadd et al., 2021). It seems likely that brain tissue may exhibit different alterations in methylation in response to inflammation that were not captured by the two DNAm inflammatory marker proxies utilised here. In contrast to the inflammatory results, a higher DNAm age acceleration in the hippocampus was found for each of the assessed measures apart from AgeAccel_Grim_. This was true both for the cortex‐specific clock as well as for the measures developed in the blood (AgeAccel_Pheno_ and EEAA) or in multiple tissues (IEAA). This consistency implies that the hippocampus may represent a region more susceptible to biological ageing than other areas of the neocortex. Age‐related decline in hippocampal volume is well established (Raz et al., 2005) and it is one of the earliest, and most profoundly, affected regions in Alzheimer's disease, suffering insidious synapse loss and neuronal cell death culminating in a substantial atrophy as the disease progresses (Braak et al., 1993). While none of the individuals included in this study had a diagnosis of Alzheimer's disease prior to their death, the hippocampus can suffer substantial deterioration before clinical dementia becomes evident and some of the participants did have evidence of amyloidosis at post mortem. The accelerated epigenetic ageing noted here is perhaps capturing the vulnerability of this region.

Equivalent to this finding, we identified a higher percentage burden of CD68^+^ microglia in the hippocampus compared with BA17. CD68 is a marker of phagocytic activity and is typically used to classify reactive microglia. Microglia are important in the maintenance of integrity and function within the central nervous system; however, aged microglia have been shown to be more responsive to pro‐inflammatory stimuli compared with the naïve cell‐type. This altered phenotype can lead to exaggerated neuro‐inflammation in response to peripheral or central immune challenges which can precipitate neuro‐toxicity, and thus, degeneration (Luo et al., 2010; Perry & Holmes, 2014). The only association identified between the DNAm signatures and microglial load was a positive association with the mean brain AgeAccel_Pheno_. However, we did not find any significant interaction between the DNAm signatures and region. The DNAm PhenoAge clock was trained on a set of nine haematological and biochemical measures that were found to optimally predict an individual's ‘phenotypic age’ including four immune cell profiles (lymphocyte percent, mean cell volume, red cell distribution width and white blood cell count) alongside CRP and albumin (Levine et al., 2018). Despite being developed on blood DNAm data, the predominantly inflammatory and immune composition of this clock may mean that AgeAccel_Pheno_ is better able to capture process associated with inflammation even in other tissues. In this regard, it may have outperformed the DNAm CRP and IL‐6 score due to the inclusion of a composite set of phenotypes, which may more accurately index systemic inflammation compared with a single inflammatory surrogate.

This study provides a rarely‐available assessment of data from blood, alongside post mortem brain tissue methylation profiles and histology from the same individuals. Alongside this, profiling DNAm in multiple regions of the brain allowed us to investigate the heterogeneity of methylation patterns within the same tissue type. This study is limited by the small number of individuals for which data was available, leading to a lack of statistical power and the potential for both type 1 and type 2 errors. We considered *p* < 0.05 as the threshold for statistical significance in the analyses. However, the following associations fail to pass a strict Bonferroni‐corrected threshold (*p* ≤ 0.001): the differences of the DNAm IL‐6 score across the brain regions, the IEAA measure being highest in the hippocampus compared with BA17 and the association of DNAm AgeAccel_Pheno_ with the CD68+ microglia burden. This, again, highlights that the results presented here should be taken as preliminary patterns until analyses can be repeated in larger sample sizes. Different array platforms were utilised for the methylation profiling of the blood and brain tissue, and, as such, the QC pipelines were slightly different which may have influenced downstream analysis. In regard to the microglial burdening, we used only one antibody (CD68) which limited definitive identification of labelled cells as parenchymal microglia. CD68 stains the lysosomes of ostensibly reactive microglia; however, the antibody can additionally stain infiltrating macrophages. Capturing both the microglia and macrophage burden still provides a relevant read‐out of the cellular inflammatory status; however, further characterisation of the microglial phenotype, including generating a reactive:total ratio would be desirable to glean a better understanding of their specific relationship to DNAm signatures. Further to this, the burden metric used to quantify microglia could reflect differences in sizes of the cells as well as in total numbers. An additional aspect to bear in mind when utilising post mortem tissue in methylation studies is the stability of global DNAm following death and the biological implications of this (Pidsley & Mill, 2011; Sjöholm et al., 2018). We attempted to account for the potential impact of this by adjusting analyses for post mortem intervals; however as post mortem changes in DNAm are not yet well characterised it cannot be ruled out that this confounded results. Similarly, cause‐of‐death may have impacted inflammatory profiles within the brain so we cannot rule out this influenced results. Finally, post mortem studies will always be retrospective in nature, rendering it impossible to discern causal or consequential events.

In summary, using a well‐characterised cohort of 14 individuals, we identified divergent correlations between the blood and brain in DNAm inflammation‐related and age acceleration measures depending on region assessed. The hippocampus was found to display the highest DNAm age acceleration in four out of five assessed measures, potentially reflecting its inherent susceptibility to biological ageing and pathological processes compared with other cortical regions. The hippocampus additionally showed the highest burden of reactive microglia. Whilst an accelerated DNAm PhenoAge associated with an elevated microglial load across the brain, no region‐specific associations were identified. Our results provide some initial indications of the blood–brain relationships in DNAm patterns and how these relate to central processes; however further work is needed to verify these results in larger sample sizes and to investigate how these patterns associate with cognitive function and neurodegenerative disease.

## CONFLICT OF INTEREST

LM and REM have received payment from Illumina for presentations. REM is an advisor to the Epigenetic Clock Development Foundation. TS‐J serves as a scientific advisor to Cognition Therapeutics, receives collaborative funding from an anonymous pharmaceutical company, and received honoraria for presentations at commercial and academic institutes unrelated to this paper. All other authors declare no competing interests.

## AUTHOR CONTRIBUTIONS

Conception and design: AJS, REM and TSJ. Data analysis: AJS, DAG and DLMcC. Drafting the article: AJS. Data preparation: GS, NW, SMcC, LM, PR and AMT. Revision of the article: all authors. All authors read and approved the final manuscript.

5

### PEER REVIEW

The peer review history for this article is available at https://publons.com/publon/10.1111/ejn.15661.

### DATA AVAILABILTIY STATEMENT

LBC1936 data are available on request from the Lothian Birth Cohort Study, University of Edinburgh (simon.cox@ed.ac.uk). LBC1936 data are not publicly available due to them containing information that could compromise participant consent and confidentiality.

## Supporting information


**Table S1.** *Post‐mortem* details for the Lothian Birth Cohort 1936 participants.
**Table S2.** The DNAm CRP score probes and their coefficients. The CpG highlighted in bold was not available on the EPIC array and was omitted from analyses.
**Table S3.** The DNAm IL‐6 score probes and their coefficients. CpGs highlighted in bold were not in the original DNAm IL‐6 score (derived from LBC1936 data inclusive of those who had donated *post‐mortem* brain tissue: see methods section).
**Table S4.** The differences in the DNAm CRP and IL‐6 scores over the brain regions. BA17 was used as the reference. Associations with P < 0.05 are highlighted in bold.
DNAm = DNA methylation; CRP=C‐reactive protein; IL‐6 = interleukin‐6; BA = Brodmann area.
**Table S5.** The differences in the DNAm age acceleration measures over the brain regions.
**Table S6.** Associations between DNAm age acceleration and inflammation scores in the blood and the brain and CD68 + microglial burdens.
**Figure S1.** Spearman correlations between the last blood DNAm CRP score and the DNAm CRP score in each of the five brain regions assessed. The correlation coefficients and 95% confidence intervals are presented in the table.
**Figure S2.** Spearman correlations between the last blood DNAm IL‐6 score and the DNAm IL‐6 score in each of the five brain regions assessed. The correlation coefficients and 95% confidence intervals are presented in the table.
**Figure S3.** Spearman correlations between the last blood IEAA and IEAA in each of the five brain regions assessed. The correlation coefficients and 95% confidence intervals are presented in the table.
**Figure S4.** Spearman correlations between the last blood EEAA and EEAA in each of the five brain regions assessed. The correlation coefficients and 95% confidence intervals are presented in the table.
**Figure S5.** Spearman correlations between the last blood AgeAccelGrim and AgeAccelGrim in each of the five brain regions assessed. The correlation coefficients and 95% confidence intervals are presented in the table.
**Figure S6.** Spearman correlations between the last blood AgeAccelPheno and AgeAccelPheno in each of the five brain regions assessed. The correlation coefficients and 95% confidence intervals are presented in the table.
**Figure S7.** Spearman correlations between the epigenetic age acceleration measures and DNAm inflammatory measures within the blood.
**Figure S8.** Spearman correlations between the epigenetic age acceleration measures and DNAm inflammatory measures within the brain (mean across all five regions).
**Figure S9.** Exemplar images of the staining in each of the brain regions analysed. Scale bar = 150 μm.

## References

[ejn15661-bib-0001] Amor, S. , Puentes, F. , Baker, D. , & van der Valk, P. (2010). Inflammation in neurodegenerative diseases. Immunology, 129, 154–169. 10.1111/j.1365-2567.2009.03225.x 20561356 PMC2814458

[ejn15661-bib-0002] Barker, E. D. , Cecil, C. A. M. , Walton, E. , Houtepen, L. C. , O'Connor, T. G. , Danese, A. , Jaffee, S. R. , Jensen, S. K. G. , Pariante, C. , McArdle, W. , Gaunt, T. R. , Relton, C. L. , & Roberts, S. (2018). Inflammation‐related epigenetic risk and child and adolescent mental health: A prospective study from pregnancy to middle adolescence. Development and Psychopathology, 30, 1145–1156. 10.1017/S0954579418000330 30068408 PMC7612578

[ejn15661-bib-0003] Beck, S. , & Rakyan, V. K. (2008). The methylome: Approaches for global DNA methylation profiling. Trends in Genetics, 24(5), 231–237. 10.1016/j.tig.2008.01.006 18325624

[ejn15661-bib-0004] Beydoun, M. A. , Shaked, D. , Tajuddin, S. M. , Weiss, J. , Evans, M. K. , & Zonderman, A. B. (2020). Accelerated epigenetic age and cognitive decline among urban‐dwelling adults. Neurology, 94(6), e613–e625. 10.1212/WNL.0000000000008756 31879275 PMC7136071

[ejn15661-bib-0005] Braak, H. , Braak, E. , & Bohl, J. (1993). Staging of Alzheimer‐related cortical destruction. European Neurology, 33(6), 403–408. 10.1159/000116984 8307060

[ejn15661-bib-0006] Chuang, Y.‐H. , Paul, K. C. , Bronstein, J. M. , Bordelon, Y. , Horvath, S. , & Ritz, B. (2017). Parkinson's disease is associated with DNA methylation levels in human blood and saliva. Genome Medicine, 9(1), 76. 10.1186/s13073-017-0466-5 28851441 PMC5576382

[ejn15661-bib-0007] Cui, J. G. , Hill, J. M. , Zhao, Y. , & Lukiw, W. J. (2007). Expression of inflammatory genes in the primary visual cortex of late‐stage Alzheimer's disease. Neuroreport, 18, 115–119. 10.1097/WNR.0b013e32801198bc 17301674

[ejn15661-bib-0008] Cunningham, C. , Wilcockson, D. C. , Campion, S. , Lunnon, K. , & Perry, V. H. (2005). Central and systemic endotoxin challenges exacerbate the local inflammatory response and increase neuronal death during chronic neurodegeneration. The Journal of Neuroscience, 25(40), 9275–9284. 10.1523/JNEUROSCI.2614-05.2005 16207887 PMC6725757

[ejn15661-bib-0009] Deary, I. J. , Gow, A. J. , Taylor, M. D. , Corley, J. , Brett, C. , Wilson, V. , Campbell, H. , Whalley, L. J. , Visscher, P. M. , Porteous, D. J. , & Starr, J. M. (2007). The Lothian birth cohort 1936: A study to examine influences on cognitive ageing from age 11 to age 70 and beyond. BMC Geriatrics, 7, 28. 10.1186/1471-2318-7-28 18053258 PMC2222601

[ejn15661-bib-0010] Di Francesco, A. , Arosio, B. , Falconi, A. , Micioni Di Bonaventura, M. V. , Karimi, M. , Mari, D. , Casati, M. , Maccarrone, M. , & DAddario, C. (2015). Global changes in DNA methylation in Alzheimers disease peripheral blood mononuclear cells. Brain, Behavior, and Immunity, 45, 139–144. 10.1016/j.bbi.2014.11.002 25452147

[ejn15661-bib-0011] El Khoury, L. Y. , Gorrie‐Stone, T. , Smart, M. , Hughes, A. , Bao, Y. , Andrayas, A. , Burrage, J. , Hannon, E. , Kumari, M. , Mill, J. , & Schalkwyk, L. C. (2019). Systematic underestimation of the epigenetic clock and age acceleration in older subjects. Genome Biology, 20(1), 283. 10.1186/s13059-019-1810-4 31847916 PMC6915902

[ejn15661-bib-0012] Elwood, E. , Lim, Z. , Naveed, H. , & Galea, I. (2017). The effect of systemic inflammation on human brain barrier function. Brain, Behavior, and Immunity, 62, 35–40. 10.1016/j.bbi.2016.10.020 27810376 PMC5380128

[ejn15661-bib-0013] Franceschi, C. , Bonafè, M. , Valensin, S. , Olivieri, F. , De Luca, M. , Ottaviani, E. , & De Benedictis, G. (2000). Inflamm‐aging. An evolutionary perspective on immunosenescence. Annals of the new York Academy of Sciences, 908(1), 244–254. 10.1111/j.1749-6632.2000.tb06651.x 10911963

[ejn15661-bib-0014] Gadd, D. A. , Stevenson, A. J. , Hillary, R. F. , McCartney, D. L. , Wrobel, N. , McCafferty, S. , Murphy, L. , Russ, T. C. , Harris, S. E. , Redmond, P. , Taylor, A. M. , Smith, C. , Rose, J. , Millar, T. , Spires‐Jones, T. L. , Cox, S. R. , & Marioni, R. E. (2021). Epigenetic predictors of lifestyle traits applied to the blood and brain. Brain Communications, 3(2), fcab082. 10.1093/braincomms/fcab082 34041477 PMC8134833

[ejn15661-bib-0015] Gonzalez‐Jaramillo, V. , Portilla‐Fernandez, E. , Glisic, M. , Voortman, T. , Ghanbari, M. , Bramer, W. , Chowdhury, R. , Nijsten, T. , Dehghan, A. , Franco, O.H. & Nano, J. (2019) Epigenetics and inflammatory markers: A systematic review of the current evidence. International Journal of Inflammation, 2019, 6273680. 10.1155/2019/6273680 31205673 PMC6530203

[ejn15661-bib-0016] Guintivano, J. , Aryee, M. J. , & Kaminsky, Z. A. (2013). A cell epigenotype specific model for the correction of brain cellular heterogeneity bias and its application to age, brain region and major depression. Epigenetics, 8, 290–302. 10.4161/epi.23924 23426267 PMC3669121

[ejn15661-bib-0017] Hannon, E. , Lunnon, K. , Schalkwyk, L. , & Mill, J. (2015). Interindividual methylomic variation across blood, cortex, and cerebellum: Implications for epigenetic studies of neurological and neuropsychiatric phenotypes. Epigenetics, 10(11), 1024–1032. 10.1080/15592294.2015.1100786 26457534 PMC4844197

[ejn15661-bib-0018] Hannum, G. , Guinney, J. , Zhao, L. , Zhang, L. , Hughes, G. , Sadda, S. , Klotzle, B. , Bibikova, M. , Fan, J.‐B. , & Gao, Y. (2013). Genome‐wide methylation profiles reveal quantitative views of human aging rates. Molecular Cell, 49, 359–367. 10.1016/j.molcel.2012.10.016 23177740 PMC3780611

[ejn15661-bib-0019] Hillary, R. F. , Stevenson, A. J. , Cox, S. R. , McCartney, D. L. , Harris, S. E. , Seeboth, A. , Higham, J. , Sproul, D. , Taylor, A. M. , Redmond, P. , Corley, J. , Pattie, A. , Hernández, M. D. C. V. , Muñoz‐Maniega, S. , Bastin, M. E. , Wardlaw, J. M. , Horvath, S. , Ritchie, C. W. , Spires‐Jones, T. L. , … Marioni, R. E. (2019). An epigenetic predictor of death captures multi‐modal measures of brain health. Molecular Psychiatry, 26, 3806–3816. 31796892 10.1038/s41380-019-0616-9 PMC8550950

[ejn15661-bib-0020] Horvath, S. (2013). DNA methylation age of human tissues and cell types. Genome Biology, 14(10), R115. 10.1186/gb-2013-14-10-r115 24138928 PMC4015143

[ejn15661-bib-0021] Jaenisch, R. , & Bird, A. (2003). Epigenetic regulation of gene expression: How the genome integrates intrinsic and environmental signals. Nature Genetics, 33(S3), 245–254. 10.1038/ng1089 12610534

[ejn15661-bib-0022] Kaneshwaran, K. , Olah, M. , Tasaki, S. , Yu, L. , Bradshaw, E. M. , Schneider, J. A. , Buchman, A. S. , Bennett, D. A. , De Jager, P. L. , & Lim, A. S. P. (2019). Sleep fragmentation, microglial aging, and cognitive impairment in adults with and without Alzheimer's dementia. Science Advances, 5(12), eaax7331. 10.1126/sciadv.aax7331 31844665 PMC6905859

[ejn15661-bib-0023] Keller, J. N. (2006). Age‐related neuropathology, cognitive decline, and Alzheimer's disease. Ageing Research Reviews, 5, 1–13. 10.1016/j.arr.2005.06.002 16084778

[ejn15661-bib-0024] Levine, M. E. , Lu, A. T. , Quach, A. , Chen, B. H. , Assimes, T. L. , Bandinelli, S. , Hou, L. , Baccarelli, A. A. , Stewart, J. D. , Li, Y. , Whitsel, E. A. , Wilson, J. G. , Reiner, A. P. , Aviv, A. , Lohman, K. , Liu, Y. , Ferrucci, L. , & Horvath, S. (2018). An epigenetic biomarker of aging for lifespan and healthspan. Aging, 10(4), 573–591. 10.18632/aging.101414 29676998 PMC5940111

[ejn15661-bib-0025] Ligthart, S. , Marzi, C. , Aslibekyan, S. , Mendelson, M. M. , Conneely, K. N. , Tanaka, T. , Colicino, E. , Waite, L. L. , Joehanes, R. , Guan, W. , Brody, J. A. , Elks, C. , Marioni, R. , Jhun, M. A. , Agha, G. , Bressler, J. , Ward‐Caviness, C. K. , Chen, B. H. , Huan, T. , … Disease, C.e.o. C. H. (2016). DNA methylation signatures of chronic low‐grade inflammation are associated with complex diseases. Genome Biology, 17, 255. 10.1186/s13059-016-1119-5 27955697 PMC5151130

[ejn15661-bib-0026] Liu, C. , Marioni, R. E. , Hedman, Å. K. , Pfeiffer, L. , Tsai, P. C. , Reynolds, L. M. , Just, A. C. , Duan, Q. , Boer, C. G. , Tanaka, T. , Elks, C. E. , Aslibekyan, S. , Brody, J. A. , Kühnel, B. , Herder, C. , Almli, L. M. , Zhi, D. , Wang, Y. , Huan, T. , … Levy, D. (2018). A DNA methylation biomarker of alcohol consumption. Molecular Psychiatry, 23, 422–433. 10.1038/mp.2016.192 27843151 PMC5575985

[ejn15661-bib-0027] Lu, A. T. , Quach, A. , Wilson, J. G. , Reiner, A. P. , Aviv, A. , Raj, K. , Hou, L. , Baccarelli, A. A. , Li, Y. , Stewart, J. D. , Whitsel, E. A. , Assimes, T. L. , Ferrucci, L. , & Horvath, S. (2019). DNA methylation GrimAge strongly predicts lifespan and healthspan. Aging, 11, 303–327. 10.18632/aging.101684 30669119 PMC6366976

[ejn15661-bib-0028] Luo, X.‐G. , Ding, J.‐Q. , & Chen, S.‐D. (2010). Microglia in the aging brain: Relevance to neurodegeneration. Molecular Neurodegeneration, 5, 12. 10.1186/1750-1326-5-12 20334662 PMC2852379

[ejn15661-bib-0029] Marioni, R. E. , Shah, S. , McRae, A. F. , Ritchie, S. J. , Muniz‐Terrera, G. , Harris, S. E. , Gibson, J. , Redmond, P. , Cox, S. R. , Pattie, A. , Corley, J. , Taylor, A. , Murphy, L. , Starr, J. M. , Horvath, S. , Visscher, P. M. , Wray, N. R. , & Deary, I. J. (2015). The epigenetic clock is correlated with physical and cognitive fitness in the Lothian birth cohort 1936. International Journal of Epidemiology, 44, 1388–1396. 10.1093/ije/dyu277 25617346 PMC4588858

[ejn15661-bib-0030] McCartney, D. L. , Hillary, R. F. , Conole, E. L. S. , Banos, D. T. , Gadd, D. A. , Walker, R. M. , Nangle, C. , Flaig, R. , Campbell, A. , Murray, A. D. , Maniega, S. M. , Valdés‐Hernández, M. D. C. , Harris, M. A. , Bastin, M. E. , Wardlaw, J. M. , Harris, S. E. , Porteous, D. J. , Tucker‐Drob, E. M. , McIntosh, A. M. , … Marioni, R. E. (2022). Blood‐based epigenome‐wide analyses of cognitive abilities. Genome Biology, 23(1), 26. 10.1186/s13059-021-02596-5 35039062 PMC8762878

[ejn15661-bib-0031] McCartney, D. L. , Stevenson, A. J. , Hillary, R. F. , Walker, R. M. , Bermingham, M. L. , Morris, S. W. , Clarke, T.‐K. , Campbell, A. , Murray, A. D. , Whalley, H. C. , Porteous, D. J. , Visscher, P. M. , McIntosh, A. M. , Evans, K. L. , Deary, I. J. , & Marioni, R. E. (2018). Epigenetic signatures of starting and stopping smoking. eBioMedicine, 37, 214–220. 10.1016/j.ebiom.2018.10.051 30389506 PMC6286188

[ejn15661-bib-0032] Mendizabal, I. , & Yi, S. V. (2016). Whole‐genome bisulfite sequencing maps from multiple human tissues reveal novel CpG islands associated with tissue‐specific regulation. Human Molecular Genetics, 25(1), 69–82. 10.1093/hmg/ddv449 26512062 PMC4690492

[ejn15661-bib-0033] Norden, D. M. , & Godbout, J. P. (2013). Review: Microglia of the aged brain: Primed to be activated and resistant to regulation. Neuropathology and Applied Neurobiology, 39, 19–34. 10.1111/j.1365-2990.2012.01306.x 23039106 PMC3553257

[ejn15661-bib-0034] Perry, V. H. , & Holmes, C. (2014). Microglial priming in neurodegenerative disease. Nature Reviews. Neurology, 10, 217–224. 10.1038/nrneurol.2014.38 24638131

[ejn15661-bib-0035] Pidsley, R. , & Mill, J. (2011). Epigenetic studies of psychosis: Current findings, methodological approaches, and implications for postmortem research. Biological Psychiatry, 69, 146–156. 10.1016/j.biopsych.2010.03.029 20510393

[ejn15661-bib-0036] Pidsley, R. , Y Wong, C. C. , Volta, M. , Lunnon, K. , Mill, J. , & Schalkwyk, L. C. (2013). A data‐driven approach to preprocessing Illumina 450K methylation array data. BMC Genomics, 14, 293. 10.1186/1471-2164-14-293 23631413 PMC3769145

[ejn15661-bib-0037] Raz, N. , Lindenberger, U. , Rodrigue, K. M. , Kennedy, K. M. , Head, D. , Williamson, A. , Dahle, C. , Gerstorf, D. , & Acker, J. D. (2005). Regional brain changes in aging healthy adults: general trends, individual differences and modifiers. Cerebral Cortex (New York, N.Y.: 1991), 15, 1676–1689. 15703252 10.1093/cercor/bhi044

[ejn15661-bib-0038] Samarasekera, N. , Al‐Shahi Salman, R. , Huitinga, I. , Klioueva, N. , McLean, C. A. , Kretzschmar, H. , Smith, C. , & Ironside, J. W. (2013). Brain banking for neurological disorders. Lancet Neurology, 12, 1096–1105. 10.1016/S1474-4422(13)70202-3 24074724

[ejn15661-bib-0039] Shah, S. , McRae, A. F. , Marioni, R. E. , Harris, S. E. , Gibson, J. , Henders, A. K. , Redmond, P. , Cox, S. R. , Pattie, A. , Corley, J. , Murphy, L. , Martin, N. G. , Montgomery, G. W. , Starr, J. M. , Wray, N. R. , Deary, I. J. , & Visscher, P. M. (2014). Genetic and environmental exposures constrain epigenetic drift over the human life course. Genome Research, 24(11), 1725–1733. 10.1101/gr.176933.114 25249537 PMC4216914

[ejn15661-bib-0040] Shireby, G. L. , Davies, J. P. , Francis, P. T. , Burrage, J. , Walker, E. M. , Neilson, G. W. A. , Dahir, A. , Thomas, A. J. , Love, S. , Smith, R. G. , Lunnon, K. , Kumari, M. , Schalkwyk, L. C. , Morgan, K. , Brookes, K. , Hannon, E. , & Mill, J. (2020). Recalibrating the epigenetic clock: Implications for assessing biological age in the human cortex. Brain, 143(12), 3763–3775. 33300551 10.1093/brain/awaa334 PMC7805794

[ejn15661-bib-0041] Sjöholm, L. K. , Ransome, Y. , Ekström, T. J. , & Karlsson, O. (2018). Evaluation of post‐mortem effects on global brain DNA methylation and hydroxymethylation. Basic & Clinical Pharmacology & Toxicology, 122, 208–213. 10.1111/bcpt.12875 28834189 PMC5991080

[ejn15661-bib-0043] Stevenson, A. J. , Gadd, D. A. , Hillary, R. F. , McCartney, D. L. , Campbell, A. , Walker, R. M. , Evans, K. L. , Harris, S. E. , Spires‐Jones, T. L. , McRae, A. F. , Visscher, P. M. , McIntosh, A. M. , Deary, I. J. , & Marioni, R. E. (2021). Creating and validating a DNA methylation‐based proxy for Interleukin‐6. The Journals of Gerontology: Series A, 76(12), 2284–2292. 10.1093/gerona/glab046 PMC8599002 33595649

[ejn15661-bib-0044] Stevenson, A. J. , McCartney, D. L. , Hillary, R. F. , Campbell, A. , Morris, S. W. , Bermingham, M. L. , Walker, R. M. , Evans, K. L. , Boutin, T. S. , Hayward, C. , McRae, A. F. , McColl, B. W. , Spires‐Jones, T. L. , McIntosh, A. M. , Deary, I. J. , & Marioni, R. E. (2020). Characterisation of an inflammation‐related epigenetic score and its association with cognitive ability. Clinical Epigenetics, 12, 113. 10.1186/s13148-020-00903-8 32718350 PMC7385981

[ejn15661-bib-0045] Taylor, A. M. , Pattie, A. , & Deary, I. J. (2018). Cohort profile update: The Lothian birth cohorts of 1921 and 1936. International Journal of Epidemiology, 47, 1042–1042r. 10.1093/ije/dyy022 29546429 PMC6124629

[ejn15661-bib-0046] Tzioras, M. , Easter, J. , Harris, S. , McKenzie, C.‐A. , Smith, C. , Deary, I. , Henstridge, C. , & Spires‐Jones, T. L. (2017). Assessing amyloid‐β, tau, and glial features in Lothian birth cohort 1936 participants post‐mortem. Matters, 3, e201708000003. 10.19185/matters.201708000003

[ejn15661-bib-0047] Zaimi, I. , Pei, D. , Koestler, D. C. , Marsit, C. J. , De Vivo, I. , Tworoger, S. S. , Shields, A. E. , Kelsey, K. T. , & Michaud, D. S. (2018). Variation in DNA methylation of human blood over a 1‐year period using the Illumina MethylationEPIC array. Epigenetics, 13(10–11), 1056–1071. 10.1080/15592294.2018.1530008 30270718 PMC6342169

[ejn15661-bib-0048] Zhang, Q. , Marioni, R. E. , Robinson, M. R. , Higham, J. , Sproul, D. , Wray, N. R. , Deary, I. J. , McRae, A. F. , & Visscher, P. M. (2018). Genotype effects contribute to variation in longitudinal methylome patterns in older people. Genome Medicine, 10, 75. 10.1186/s13073-018-0585-7 30348214 PMC6198530

