# Supplementary material for: A comparison of blood and brain‐derived ageing and inflammation‐related DNA methylation signatures and their association with microglial burdens
Source: Eur J Neurosci. 2022 Apr 6;56(9):5637–49. doi: 10.1111/ejn.15661 (PMC9525452; doi:10.1111/ejn.15661)

Supplementary Table 1. *Post-mortem* details for the Lothian Birth Cohort 1936 participants.

CAA=cerebral amyloid angiopathy; COPD=chronic obstructive pulmonary disease; F=female; LVSD=left ventricular systolic dysfunction; M=male; PM=*post-mortem*; SVD=small vessel disease; WM=white matter.

Brain bank number	Age at death	Sex	Interval between blood sample and death (years)	PM interval (hours)	Brain weight (g)	Brain pH	Cause of death	PM findings
BBN_19686	77	F	0.87	75	1320	6.5	·None specified	·SVD<br>·Cerebral and cerebellar microinfarcts (lacunar)<br>·CAA<br>·Braak stage I
001.26495	78	M	3.15	39	1290	6.2	·Amyloidosis<br>·Multiple Myeloma<br>·COPD	·Braak stage I
001.28402	79	M	2.29	49	1503	6.3	·Hospital acquired pneumonia<br>·Alcohol-related cirrhosis with ascites<br>·Severe LVSD<br>·COPD	·Thal phase II<br>·Mild WM pathology<br>·Moderate non-amyloid SVD<br>·Hepatic encephalopathy<br>·Braak stage I
001.28406	79	M	4.02	72	1437	6.1	·Suspected lung carcinoma<br>·Pneumonia	·Thal phase II<br>·Moderate arteriolar CAA<br>·Braak stage II<br>·MildWM pathology<br>·Mild non-amyloid SVD
001.28793	79	F	2.96	72	1219	5.9	None specified	·Thal phase I<br>·Moderate WM pathology<br>·Moderate non-amyloid SVD<br>·Braak stage II

001.28794	79	F	3.29	72	1289	5.8	·Metastatic breast cancer	·Mild WM pathology<br>·Mild non-amyloid SVD<br>·Braak stage I
001.28960	79	M	1.57	57	1301	6.1	·Chronic Lymphocytic Leukaemia<br>·Type 2 diabetes<br>·Myelofibrosis<br>·Idiopathic thrombocytopaenia purpura	·Moderate WM pathology<br>·Moderate non-amyloid SVD
001.29082	79	F	0.29	80	1339	5.9	·Metastatic carcinoma<br>·Squamous cell carcinoma lung	·Thal amyloid phase V<br>·Mild arteriolar CAA<br>·Braak stage III<br>·Mild WM pathology<br>·Mild non-amyloid SVD
001.29086	80	F	0.84	68	1468	6.2	·Intracerebral haemorrhage<br>·Hypertension	·Cerebral haemorrhage<br>·Severe WM pathology<br>·Severe non-amyloid SVD
001.31495	81	M	2.07	38	1318	5.8	·Stage IV Lung cancer<br>·Type 2 diabetes<br>·Ischaemic heart disease	·Alzheimer disease<br>·Thal phase IV<br>·Braak stage VI<br>·Mild non-amyloid SVD<br>·Mild arteriolar CAA<br>·Mild WM pathology
001.32577	81	M	1.18	74	1313	6.1	·Metastatic lung cancer<br>·COPD	·Thal phase III<br>·Braak stage II<br>·Mild WM pathology<br>·Mild non-amyloid SVD<br>·WM microinfarcts<br>·Mild arteriolar CAA

001.34131	82	M	2.61	95	1472	5.9	·Squamous cell carcinoma of the lung	·Thal amyloid phase III<br>·Severe arteriolar CAA<br>·Braak stage IV<br>·Severe WM pathology<br>·Moderate non-amyloid SVD
001.35181	82	M	2.92	49	1496	6.1	·Bronchopneumonia<br>·Malignant neoplasm of the lung<br>·Acute renal failure<br>·Vascular dementia<br>·Ischaemic heart disease	·Thal phase II<br>·Braak stage II<br>·Mild arteriolar CAA<br>·Mild WM pathology<br>·Mild non-amyloid SVD
001.35215	82	M	6.05	40	1246	6	·Bronchopneuomina<br>·Left ventricular systolic dysfunction<br>·Hypertension<br>·Parotid abscess<br>·Prostate cancer<br>·Osteoarthritis	·Braak stage I<br>·Severe WM pathology<br>·Severe non-amyloid SVD

Supplementary Table 2. The DNAm CRP score probes and their coefficients**.** The CpG highlighted in bold was not available on the EPIC array and was omitted from analyses.

CpG	***coef***
**cg06126421**	-0.0052
cg06690548	-0.0048
cg10636246	-0.0069
cg18181703	-0.0053
cg19821297	-0.0051
cg25325512	-0.0031
cg27023597	-0.005

Supplementary Table 3. The DNAm IL-6 score probes and their coefficients. CpGs highlighted in bold were not in the original DNAm IL-6 score (derived from LBC1936 data inclusive of those who had donated *post-mortem* brain tissue: see methods section).

CpG	***coef***
**cg07005055**	0.0011
cg26144437	0.0060
cg03076319	0.015
**cg11231069**	0.0034
cg05575921	-0.011
**cg08703857**	0.0034
cg21368161	0.0087
cg04642923	0.0088
cg21123519	0.0012
cg04583842	0.0051
cg24455236	0.0042
cg25323809	0.00018
cg03885055	-0.018
cg17412005	-0.0049
cg19638572	0.024
cg14965639	-0.017
**cg14230378**	0.0088
cg20789595	-0.023
cg26230601	-0.0038
cg16508480	0.0034
**cg17501210**	-0.0029
cg12503394	0.00065
cg18925601	-0.0044
cg19584649	0.0057
**cg22411165**	-0.00026
cg04366687	0.0071
cg25250132	0.017
cg04468741	0.0017
cg23729763	0.015
cg10195814	0.00060
cg03998636	-0.00071
cg20059928	-0.013
cg04928129	-0.0062
cg12929678	0.032

Supplementary Table 4. The differences in the DNAm CRP and IL-6 scores over the brain regions. BA17 was used as the reference. Associations with P<0.05 are highlighted in bold.

DNAm=DNA methylation; CRP=C-reactive protein; IL-6=interleukin-6; BA=Brodmann area.

	**β**	**SE**	**P**
DNAm CRP score			
BA20/21	-0.14	0.32	0.65
BA24	-0.42	0.33	0.21
BA46	-0.35	0.29	0.23
Hippocampus	0.34	0.31	0.27
DNAm IL-6 score			
BA20/21	-0.29	0.19	0.13
BA24	0.46	0.20	**0.024**
BA46	-0.076	0.17	0.66
Hippocampus	-0.25	0.18	0.18

Supplementary Table 5. The differences in the DNAm age acceleration measures over the brain regions.

BA17 was used as the reference. Associations with P<0.05 are highlighted in bold.

IEAA=intrinsic epigenetic age acceleration; EEAA=extrinsic epigenetic age acceleration; BA= Brodmann area.

	β	SE	P
AgeAccel$_{Cortical}$			
BA20/21	0.21	0.20	0.29
BA24	0.14	0.21	0.52
BA46	0.44	0.18	**0.022**
Hippocampus	0.90	0.19	**2.6×10^{-5}**
AgeAccel$_{Grim}$			
BA20/21	-0.56	0.29	0.055
BA24	0.52	0.29	0.085
BA46	-0.46	0.26	0.090
Hippocampus	0.46	0.28	0.10
AgeAccel$_{Pheno}$			
BA20/21	0.34	0.28	0.23
BA24	0.065	0.29	0.83
BA46	5.5×10^{-3}	0.26	0.98
Hippocampus	1.14	0.27	**1.1×10^{-4}**
IEAA			
BA20/21	0.60	0.35	0.091
BA24	0.10	0.37	0.78
BA46	0.63	0.32	0.057
Hippocampus	0.83	0.34	**0.018**
EEAA			
BA20/21	0.25	0.25	0.32
BA24	0.15	0.26	0.57
BA46	0.31	0.28	0.18
Hippocampus	0.99	0.24	**1.3×10^{-4}**

Supplementary Table 6. Associations between DNAm age acceleration and inflammation scores in the blood and the brain and CD68+ microglial burdens.

The blood variables refer to the last measurement available prior to death. The brain variables refer to the mean across all five regions. Associations with P<0.05 are highlighted in bold.

CRP=C-reactive protein, DNAm: DNA methylation; IEAA=intrinsic epigenetic age acceleration; EEAA=extrinsic epigenetic age acceleration; IL-6=interleukin-6

	**β**	**SE**	**P**
Brain			
DNAm CRP score	0.05	0.12	0.66
DNAm IL-6 score	0.02	0.13	0.89
$AgeAccel_{Cortical}$	0.17	0.16	0.29
$AgeAccel_{Grim}$	0.16	0.13	0.21
$AgeAccel_{Pheno}$	0.40	0.12	**0.002**
IEAA	0.18	0.13	0.18
EEAA	0.06	0.14	0.68
Blood			
DNAm CRP score	-0.28	0.18	0.16
DNA IL-6 score	0.32	0.17	0.10
$AgeAccel_{Grim}$	0.25	0.26	0.36
$AgeAccel_{Pheno}$	-0.17	0.21	0.45
IEAA	-0.21	0.26	0.43
EEAA	-0.25	0.19	0.21

Supplementary Figure 1. Spearman correlations between the last blood DNAm CRP score and the DNAm CRP score in each of the five brain regions assessed. The correlation coefficients and 95% confidence intervals are presented in the table.

The age at last blood measurement ranged from 75.5 to 80.2 years and the age at death ranged from 77.6 to 82.9 years.

BA=Brodmann area; DNAm=DNA methylation; CRP=C-reactive protein

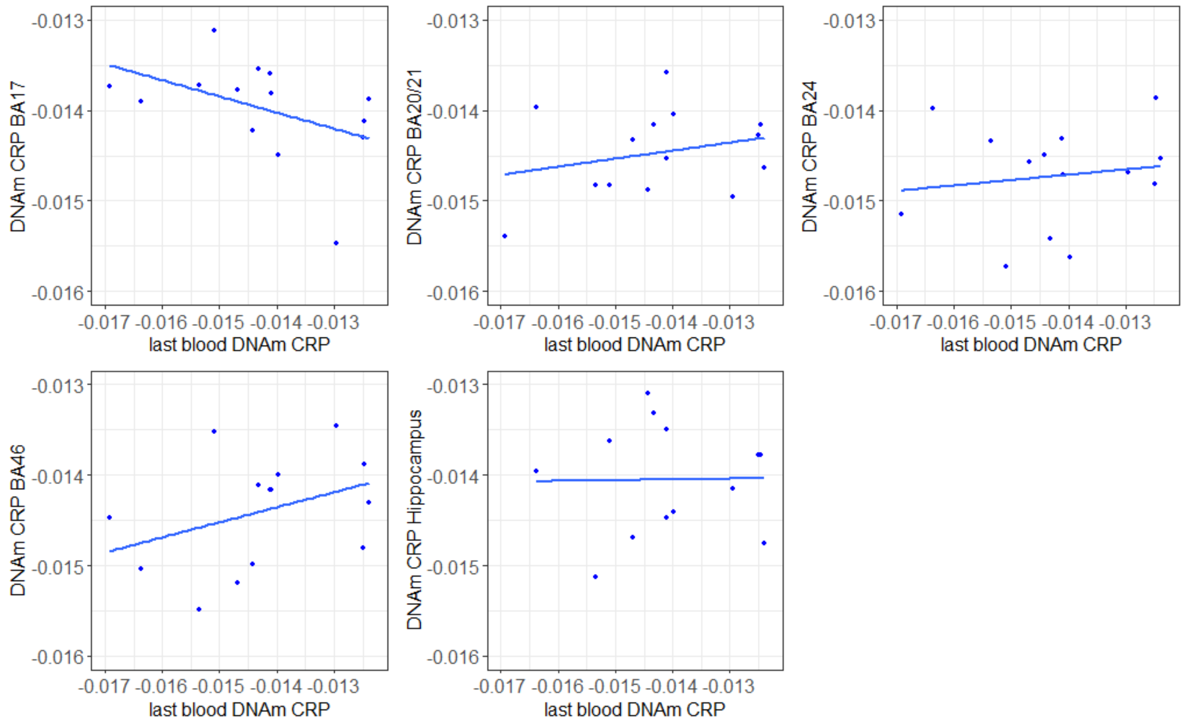

Region	r	95% confidence interval
BA17	-0.52	-0.79, 0.10
BA20/21	0.20	-0.31, 0.70
BA24	0.07	-0.42, 0.63
BA46	0.46	-0.20, 0.75
Hippocampus	-0.07	-0.54, 0.56

Supplementary Figure 2. Spearman correlations between the last blood DNAm IL-6 score and the DNAm IL-6 score in each of the five brain regions assessed. The correlation coefficients and 95% confidence intervals are presented in the table.

The age at last blood measurement ranged from 75.5 to 80.2 years and the age at death ranged from 77.6 to 82.9 years.

BA= Brodmann area; DNAm=DNA methylation; IL-6=interleukin-6

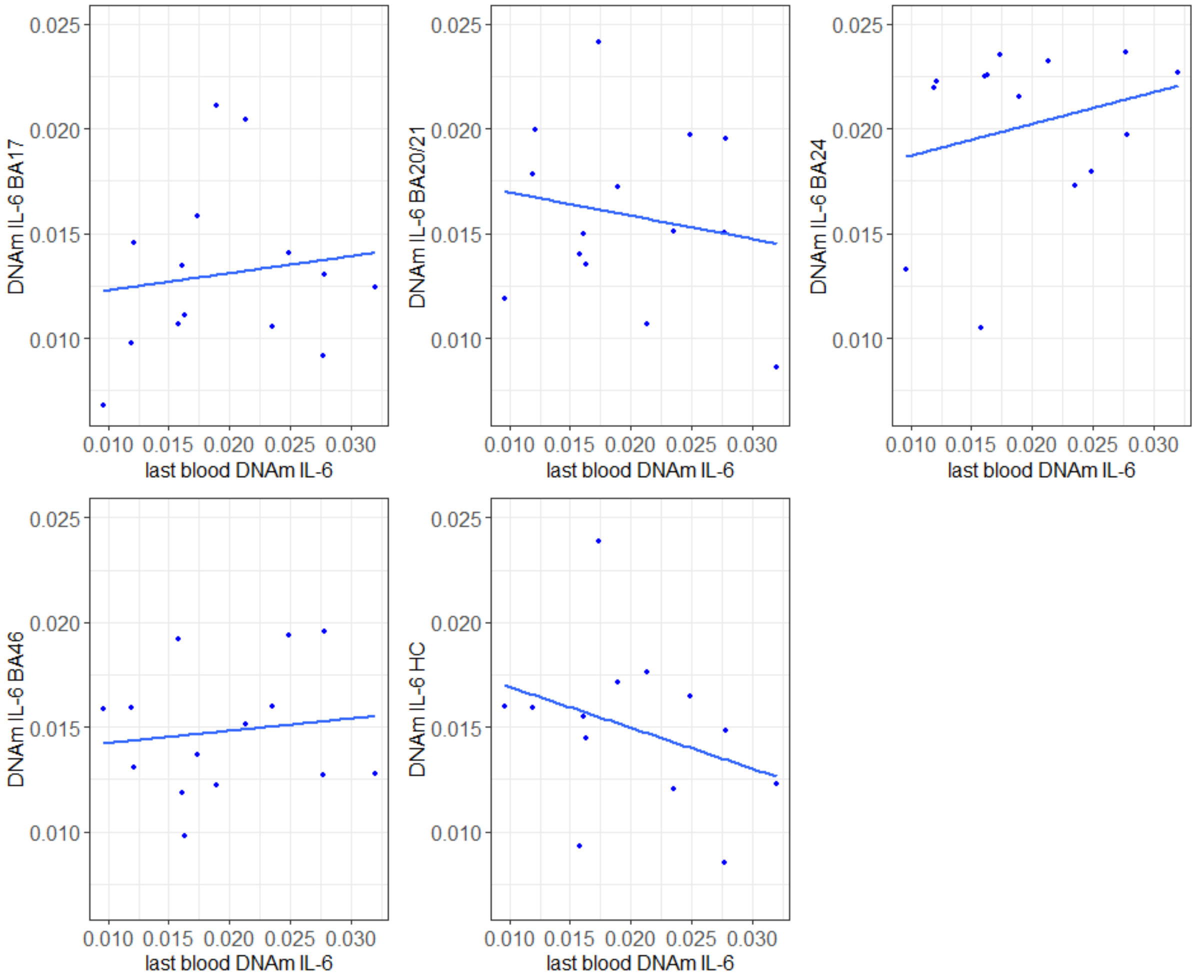

Region	r	95% confidence interval
BA17	0.14	-0.43, 0.62
BA20/21	-0.18	-0.65, 0.39
BA24	0.25	-0.32, 0.69
BA46	0.13	-0.43, 0.62
Hippocampus	-0.33	-0.75, 0.27

Supplementary Figure 3. Spearman correlations between the last blood IEAA and IEAA in each of the five brain regions assessed. The correlation coefficients and 95% confidence intervals are presented in the table.

The age at last blood measurement ranged from 75.5 to 80.2 years and the age at death ranged from 77.6 to 82.9 years.

BA= Brodmann area; IEAA=intrinsic epigenetic age acceleration

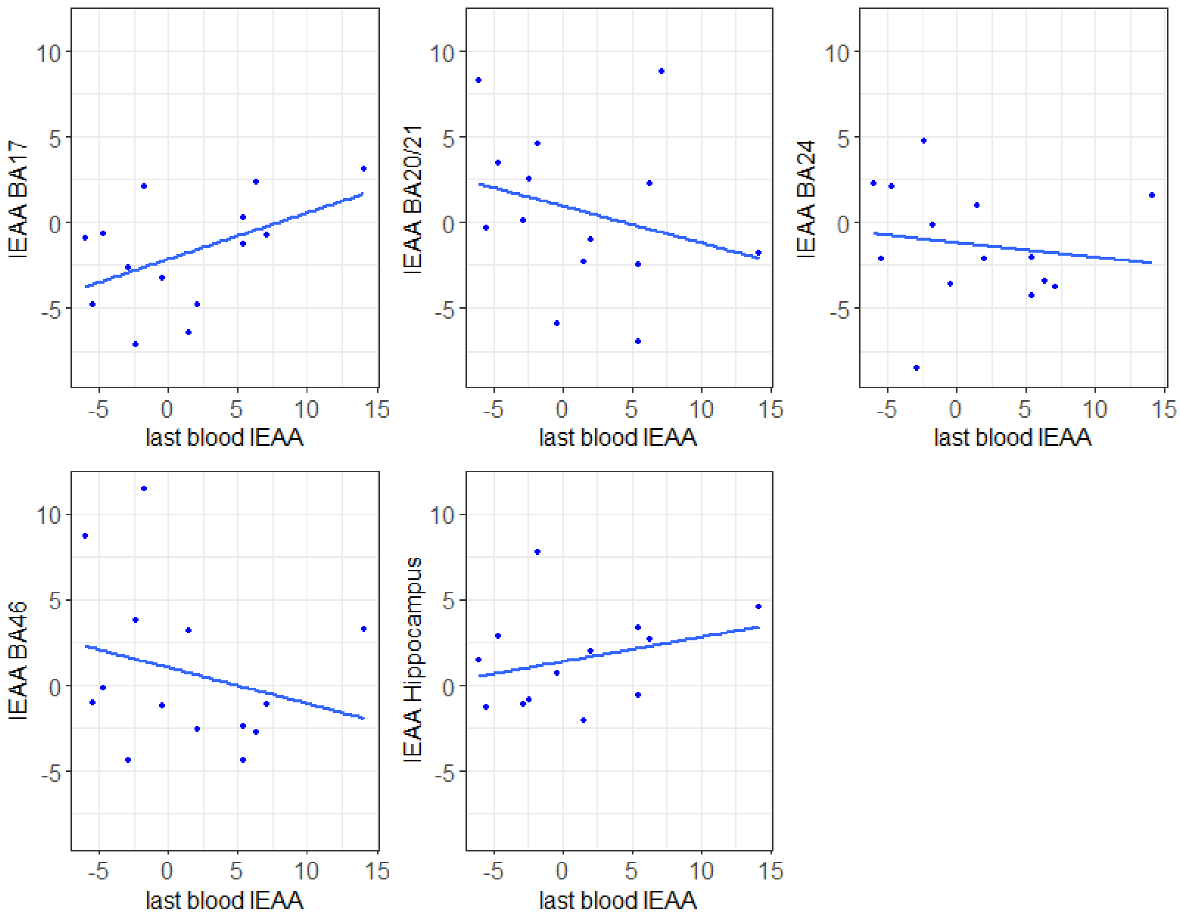

Region	r	95% confidence interval
BA17	0.49	-0.058, 0.81
BA20/21	-0.27	-0.69, 0.31
BA24	-0.14	-0.63, 0.42
BA46	-0.25	-0.69, 0.32
Hippocampus	0.30	-0.30, 0.73

Supplementary Figure 4. Spearman correlations between the last blood EEAA and EEAA in each of the five brain regions assessed. The correlation coefficients and 95% confidence intervals are presented in the table.

The age at last blood measurement ranged from 75.5 to 80.2 years and the age at death ranged from 77.6 to 82.9 years.

BA= Brodmann area; EEAA=extrinsic epigenetic age acceleration

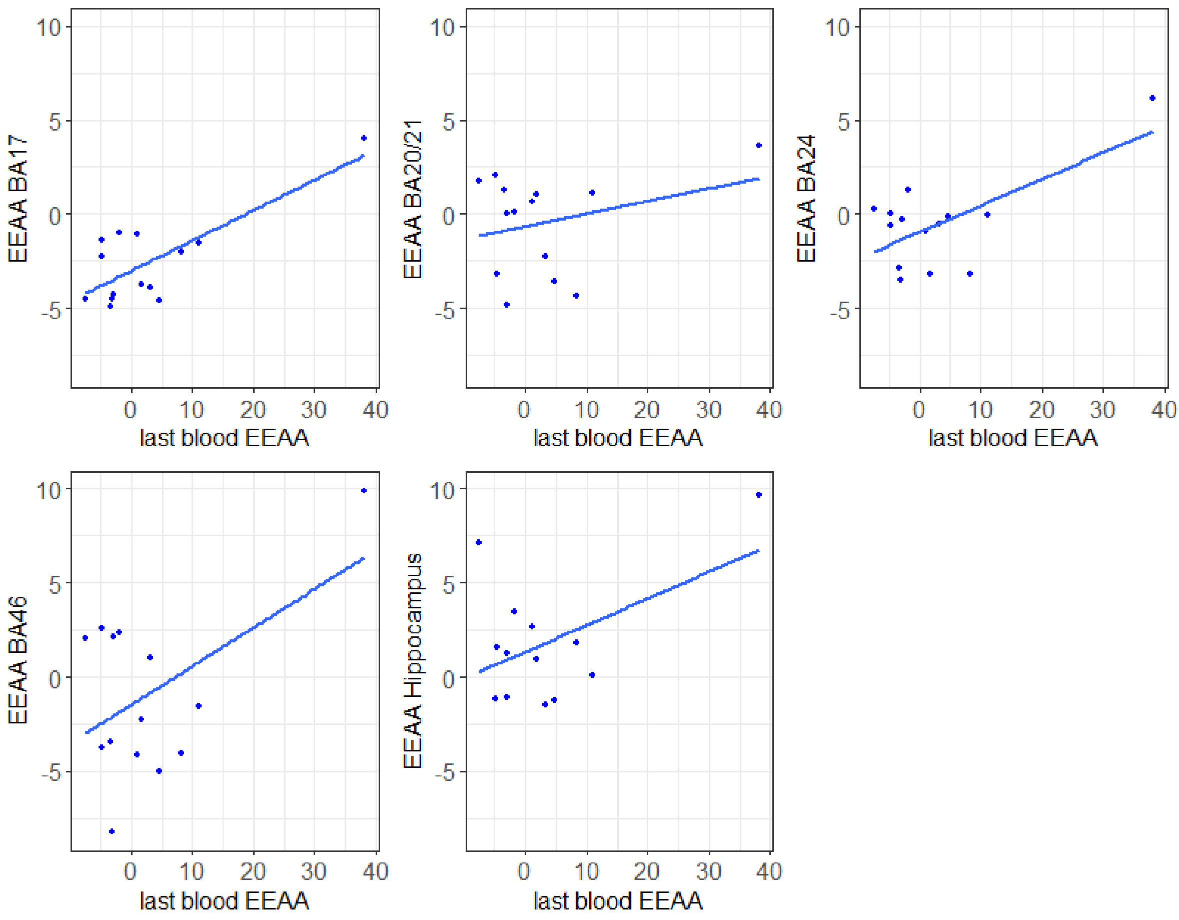

Region	r	95% confidence interval
BA17	0.78	0.42, 0.93
BA20/21	0.29	-0.29, 0.71
BA24	0.66	0.20, 0.88
BA46	0.52	0.019, 0.82
Hippocampus	0.50	-0.069, 0.82

Supplementary Figure 5. Spearman correlations between the last blood AgeAccelGrim and AgeAccelGrim in each of the five brain regions assessed. The correlation coefficients and 95% confidence intervals are presented in the table.

The age at last blood measurement ranged from 75.5 to 80.2 years and the age at death ranged from 77.6 to 82.9 years.

BA=Brodmann area

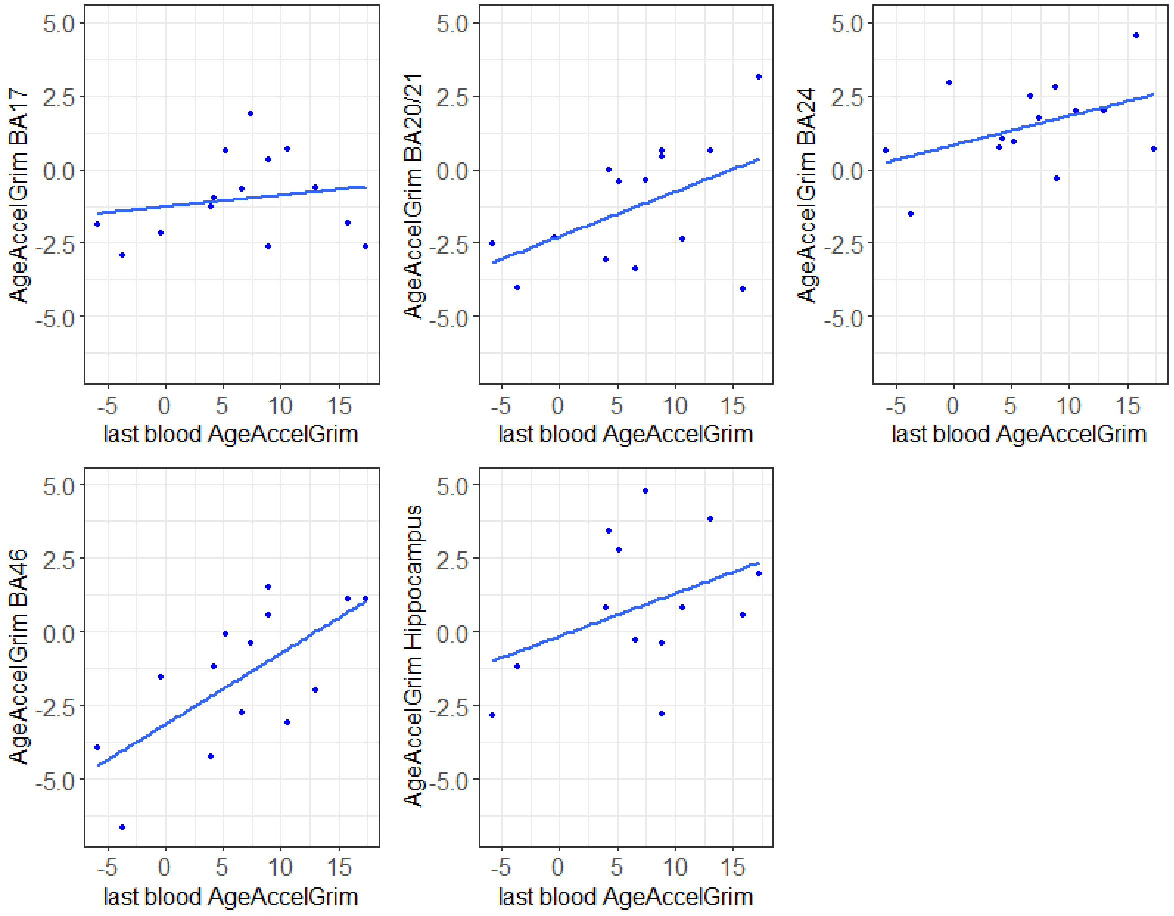

Region	r	95% confidence interval
BA17	0.18	-0.39, 0.65
BA20/21	0.48	-0.069, 0.81
BA24	0.44	-0.11, 0.79
BA46	0.68	0.23, 0.89
Hippocampus	0.40	-0.19, 0.78

Supplementary Figure 6. Spearman correlations between the last blood AgeAccelPheno and AgeAccelPheno in each of the five brain regions assessed. The correlation coefficients and 95% confidence intervals are presented in the table.

The age at last blood measurement ranged from 75.5 to 80.2 years and the age at death ranged from 77.6 to 82.9 years.

BA=Brodmann area

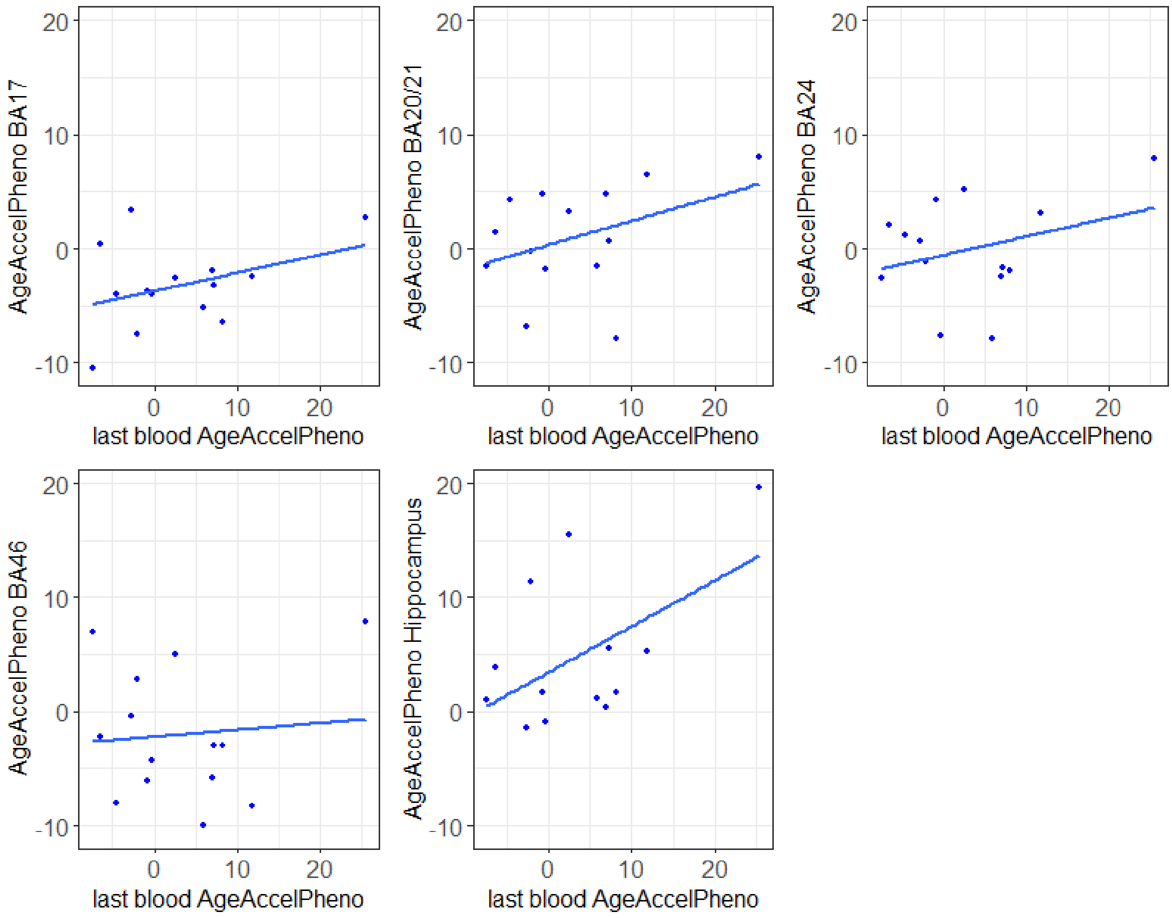

Region	r	95% confidence interval
BA17	0.37	-0.20, 0.75
BA20/21	0.39	-0.18, 0.76
BA24	0.32	-0.26, 0.73
BA46	0.09	-0.46, 0.59
Hippocampus	0.54	-0.021, 0.84

Supplementary Figure 7. Spearman correlations between the epigenetic age acceleration measures and DNAm inflammatory measures within the blood.

CRP=C-reactive protein; IL-6=interleukin-6; IEAA=intrinsic epigenetic age acceleration; EEAA=extrinsic epigenetic age acceleration

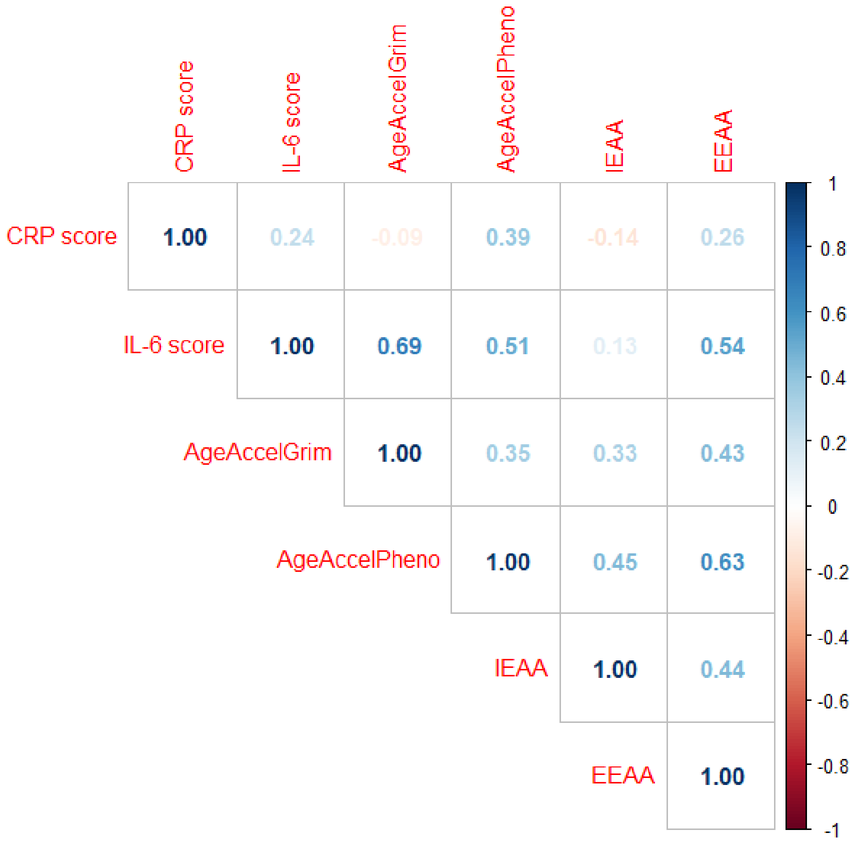

Supplementary Figure 8. Spearman correlations between the epigenetic age acceleration measures and DNAm inflammatory measures within the brain (mean across all five regions).

CRP=C-reactive protein; IL-6=interleukin-6; IEAA=intrinsic epigenetic age acceleration; EEAA=extrinsic epigenetic age acceleration

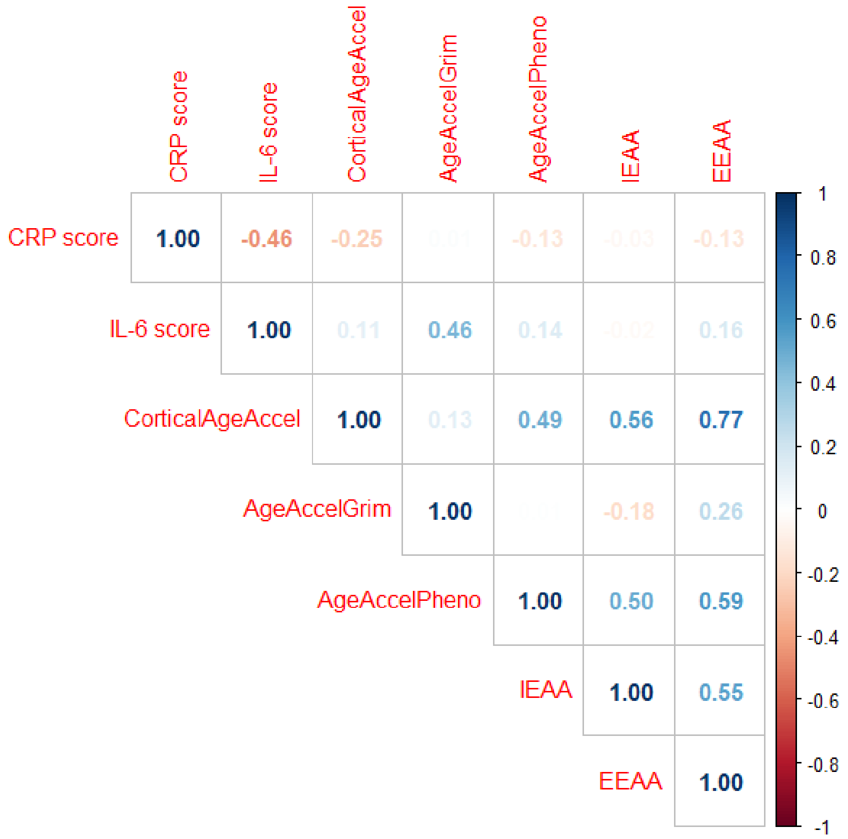

Supplementary Figure 9. Exemplar images of the staining in each of the brain regions analysed. Scale bar=150µm.

BA=Brodmann Area; HC=hippocampus.

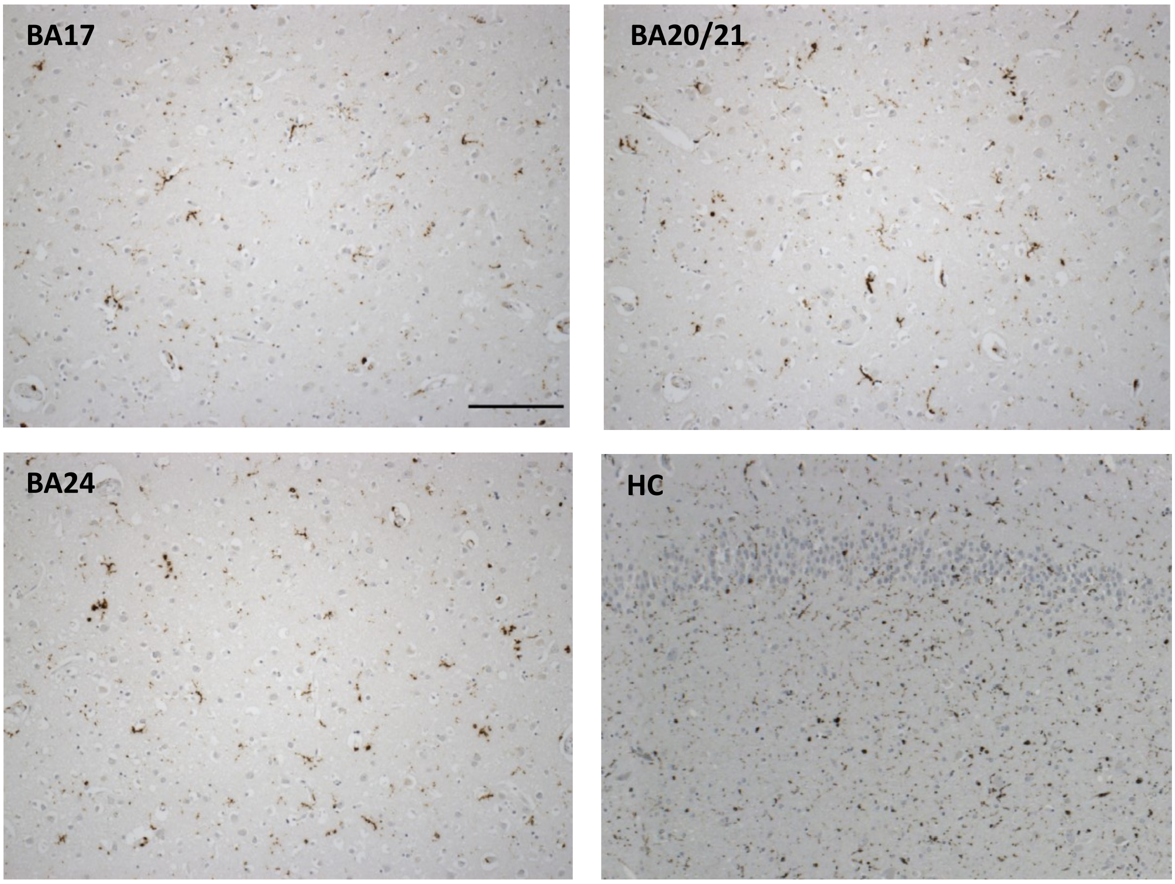

Supplement: Supplementary file 1 — Table S1. Post‐mortem details for the Lothian Birth Cohort 1936 participants. Table S2. The DNAm CRP score probes and their coefficients. The CpG highlighted in bold was not available on the EPIC array and was omitted from analyses. Table S3. The DNAm IL‐6 score probes and their coefficients. CpGs highlighted in bold were not in the original DNAm IL‐6 score (derived from LBC1936 data inclusive of those who had donated post‐mortem brain tissue: see methods section). Table S4. The differences in the DNAm CRP and IL‐6 scores over the brain regions. BA17 was used as the reference. Associations with P < 0.05 are highlighted in bold. DNAm = DNA methylation; CRP=C‐reactive protein; IL‐6 = interleukin‐6; BA = Brodmann area. Table S5. The differences in the DNAm age acceleration measures over the brain regions. Table S6. Associations between DNAm age acceleration and inflammation scores in the blood and the brain and CD68 + microglial burdens. Figure S1. Spearman correlations between the last blood DNAm CRP score and the DNAm CRP score in each of the five brain regions assessed. The correlation coefficients and 95% confidence intervals are presented in the table. Figure S2. Spearman correlations between the last blood DNAm IL‐6 score and the DNAm IL‐6 score in each of the five brain regions assessed. The correlation coefficients and 95% confidence intervals are presented in the table. Figure S3. Spearman correlations between the last blood IEAA and IEAA in each of the five brain regions assessed. The correlation coefficients and 95% confidence intervals are presented in the table. Figure S4. Spearman correlations between the last blood EEAA and EEAA in each of the five brain regions assessed. The correlation coefficients and 95% confidence intervals are presented in the table. Figure S5. Spearman correlations between the last blood AgeAccelGrim and AgeAccelGrim in each of the five brain regions assessed. The correlation coefficients and 95% confidence intervals are presented [file EJN-56-5637-s001.pdf]
